# Motivations of women in Uganda living with rheumatic heart disease: A mixed methods study of experiences in stigma, childbearing, anticoagulation, and contraception

**DOI:** 10.1371/journal.pone.0194030

**Published:** 2018-03-28

**Authors:** Andrew Y. Chang, Juliet Nabbaale, Haddy Nalubwama, Emmy Okello, Isaac Ssinabulya, Christopher T. Longenecker, Allison R. Webel

**Affiliations:** 1 Department of Medicine, Stanford University, Stanford, California, United States of America; 2 Uganda Heart Institute, Mulago Hospital, Kampala, Uganda; 3 University Hospitals Harrington Heart & Vascular Institute, Case Western Reserve University, Cleveland, Ohio, United States of America; 4 School of Public Health, Makerere University, Kampala, Uganda; 5 Frances Payne Bolton School of Nursing, Case Western Reserve University, Cleveland, Ohio, United States of America; University of Washington, UNITED STATES

## Abstract

**Background:**

Rheumatic heart disease (RHD) is a leading cause of premature mortality in low- and middle-income countries (LMICs). Women of reproductive age are a unique and vulnerable group of RHD patients, due to increased risk of cardiovascular complications and death during pregnancy. Yet, less than 5% of women of childbearing age with RHD in LMICs use contraceptives, and one in five pregnant women with RHD take warfarin despite known teratogenicity. It is unclear whether this suboptimal contraception and anticoagulant use during pregnancy is due to lack of health system resources, limited health literacy, or social pressure to bear children.

**Methods:**

We conducted a mixed methods study of 75 women living with RHD in Uganda. Questionnaires were administered to 50 patients. Transcripts from three focus groups with 25 participants were analyzed using qualitative description methodology.

**Results:**

Several themes emerged from the focus groups, including pregnancy as a calculated risk; misconceptions about side-effects of contraceptives and anticoagulation; reproductive decision-making control by male partners, in-laws, or physicians; abandonment of patients by male partners; and considerable stigma against heart disease patients for both their reproductive and financial limitations (often worse than that directed against HIV patients). All questionnaire respondents were told by physicians that their hearts were not strong enough to support a pregnancy. Only 14% used contraception while taking warfarin. All participants felt that society would look poorly on a woman who cannot have children due to a heart condition.

**Conclusions:**

To our knowledge, this is the first qualitative study of female RHD patients and their attitudes toward cardiovascular disorders and reproduction. Our results suggest that health programs targeting heart disease in LMICs must pay special attention to the needs of women of childbearing age. There are opportunities for improved family/societal education programs and community engagement, leading to better outcomes and patient empowerment.

## Introduction

Rheumatic heart disease (RHD) is one of the leading causes of premature morbidity and mortality in low- and middle-income countries (LMICs). Estimates suggest that up to 1.4 million deaths a year could be attributed to the disease [1]. RHD is an incurable chronic condition with life-threatening and potentially catastrophic health sequelae such as stroke, atrial fibrillation, and heart failure. As an illness often contracted in childhood, RHD affects people at a younger age than many other noncommunicable diseases (NCDs), resulting in excess loss of quality of life and economic potential over the lifetime of the patient [2]. Treatments to mitigate the progression of these complications of RHD exist, but they are not without their adverse effects. For example, oral anticoagulant medications such as warfarin reduce the risk of stroke and thromboembolic events in patients with atrial fibrillation or mechanical heart valve replacement, but predispose those taking them to significant bleeding events [3]. Management of these therapies is more difficult in resource-poor settings, where close monitoring and regular follow up may be a challenge.

Women of reproductive age are a particularly vulnerable group of RHD patients, since the disease places them at greater risk of complications during pregnancy [4–11]. Pre-existing heart disease in pregnant women may represent the single greatest risk factor for obstetric mortality [12–14]. Further complicating the issue for women with RHD who develop atrial fibrillation or undergo mechanical valve replacement, is that warfarin therapy—used to reduce their stroke and thromboembolism risk—is a known teratogen and increases the risk of miscarriage and maternal hemorrhage [3,15]. Heparin-based medications can be used to decrease the risk of fetal injury, but are too expensive and technically difficult to be used in many low-resource settings.

Results from a registry of RHD patients in LMICs recently found that 21% of enrolled pregnant women were taking warfarin at the time of the study [16]. Furthermore, only 3.6% of women of childbearing age in the cohort were taking contraceptive medications. In certain conditions (e.g. after 12 weeks of gestation), and under close monitoring, warfarin can be utilized during pregnancy. Yet there is potential for inappropriate usage or dosing, particularly during embryogenesis (at 6–12 weeks gestation) and in the immediate pre-delivery period (due to increased risk of obstetric hemorrhage during and after childbirth) [3]. If present, these circumstances could represent gaps in appropriate care. It is unclear if such situations including both lack of contraception and inappropriate continuation of warfarin during pregnancy would be due to lack of health system resources, limited patient health literacy, or social pressures placed on young women to bear children. To better understand these factors influencing attitudes towards reproductive control and cardiovascular disease, our group conducted a mixed methods study of 75 women living with RHD in Uganda with a combination of questionnaires and directed focus groups.

## Materials and methods

### Study design

This mixed methods study consisted of two parts: A questionnaire answered by 50 patients and a series of three focus groups of 8 to 9 patients (total 25 subjects). The questionnaires were administered by trained nurses following clinic appointments at the Uganda Heart Institute (UHI) in Kampala, Uganda. Participants who were illiterate could respond to questions verbally and have their answers recorded on the questionnaire by a research team member. Surveys were available in both English and Luganda. The focus groups were led by H.N. (a female Ugandan social worker with prior experience as a qualitative researcher and focus group facilitator), who utilized a semi-structured focus group guide designed to elicit participant experiences and opinions on reproduction, chronic disease, and therapy (S1 Protocol). The structure and content of the focus group guide was based on the socioecological model of health [17]. Focus group sessions conducted in Luganda were digitally recorded. The recordings were transcribed verbatim into English by H.N. (S3 Appendix).

### Study recruitment and ethical considerations

All participants in the study were women of reproductive age (15 to 59 years of age) carrying a clinical or echocardiographic diagnosis of RHD. Fifty patients seen at the UHI with a history of RHD were selected via consecutive sampling to complete the questionnaire. A total of 25 patients who are part of the RHD registry at UHI were selected via quota sampling to participate in the focus groups, which were conducted at UHI. All participants enrolled in the study gave informed consent and their identifying information was kept confidential (S1 Appendix, S2 Appendix). Inclusion of subjects all age groups was approved of by the IRBs of all involved institutions. All procedures were approved by the institutional review boards of Makerere University, University Hospitals Cleveland Medical Center, and Stanford University (via reliance agreement).

### Data analysis

Quantitative data from the questionnaires were described using frequencies, means, percentages, and standard deviations. Qualitative data were analyzed using qualitative description (QD) methodology [18–20]. Focus group data were examined by two research team members (A.W. and A.C.) who independently coded the data, line by line, by the constant comparative method, identifying patterns and themes [21,22]. The focus group transcripts were revisited in a series of iterative steps (by authors A.W., A.C., and J.N.) to confirm the classification of codes and theoretical saturation being reached. Data were recorded, coded, and analyzed using Provalis QDA Miner (Montreal, QC), Microsoft Word (Redmond, WA), and Microsoft Excel (Redmond, WA. The final manuscript was subjected to the COREQ checklist for consolidated criteria for reporting qualitative research (S1 Checklist) [23].

## Results

### Questionnaire results

The survey participants’ age range was 15–55 (mean 32) years, most were unemployed or homemakers (63%), and 40% did not have children (Table 1). All surveyed subjects were told by a physician that their hearts were not strong enough to support a pregnancy. 58% were on warfarin, but reported low rates of birth control use while on this anticoagulation. A majority felt that being on warfarin or having a heart condition making pregnancy risky would not reduce their desire to have children. 100% of the survey participants felt that society would look poorly on a woman who cannot have children due to a heart condition (Table 2).

**Table 1:** Sociodemographic characteristics of questionnaire participants (n = 50).

Variable	Range	Mean	Standard Deviation
Age (years old)	15–55	32	11.2
Number of Children	0–10	2.3	2.5
Employment Status	• Employed: 26% (13/50) • Unemployed: 22% (11/50) • Homemaker: 52% (26/50)		

**Table 2:** Questionnaire results.

**Question 1: Has a doctor ever told you that your heart is not strong enough to support a pregnancy?**	• **Yes:** 100% (50/50) • **No:** 0% (0/50)
**Question 2: Have you ever been on a blood thinner medication called warfarin?**	• **Yes:** 58% (29/50) • **No:** 42% (21/50)
**Question 3: Were you taking a birth control medication (pills or injection) while taking warfarin?** (For those who responded yes to Question 2 above)	• **Yes:** 14% (4/29) • **No:** 86% (25/29)
**Question 4: Did you have a IUD (intrauterine device) in place while taking warfarin?** (For those who responded yes to Question 2 above)	• **Yes:** 7% (2/29) • **No:** 93% (27/29)
**Question 5: Have you ever been pregnant while taking warfarin?** (For those who responded yes to Question 2 above)	• **Yes:** 17% (5/29) • **No:** 83% (24/29)
**Question 6: Did a doctor ever explain to you that not taking a blood thinner (if you have a heart condition that requires that you take a blood thinner) increases the chance of you suffering a life-threatening stroke or blood clot?**	• **Yes:** 58% (29/50) • **No:** 42% (21/50)
**Question 7: Did a doctor ever explain to you that taking a blood thinner increases your risk of significant bleeding during pregnancy?**	• **Yes:** 56% (28/50) • **No:** 44% (22/50)
**Question 8: Did a doctor ever explain to you that taking warfarin increases the chance of birth defects in an unborn child during pregnancy?**	• **Yes:** 53% (26/49) • **No:** 47% (23/49)
**Question 9: Would being on warfarin reduce your desire to have children?**	• **Yes:** 26% (13/50) • **No:** 74% (37/50)
**Question 10: Would having a heart condition that makes pregnancy risky reduce your desire to have children?**	• **Yes:** 38% (19/50) • **No:** 62% (31/50)
**Question 11: How important is it for a woman to be able to have low-risk pregnancy?**	• Important: 42% (21/50) • Neutral: 36% (18/50) • Unimportant: 22% (11/50)
**Question 12: Do you think that society would look poorly on a woman who cannot have children due to a heart condition?**	• **Yes:** 100% (50/50) • **No:** 0% (0/50)

### Focus group results

**Fig 1:**
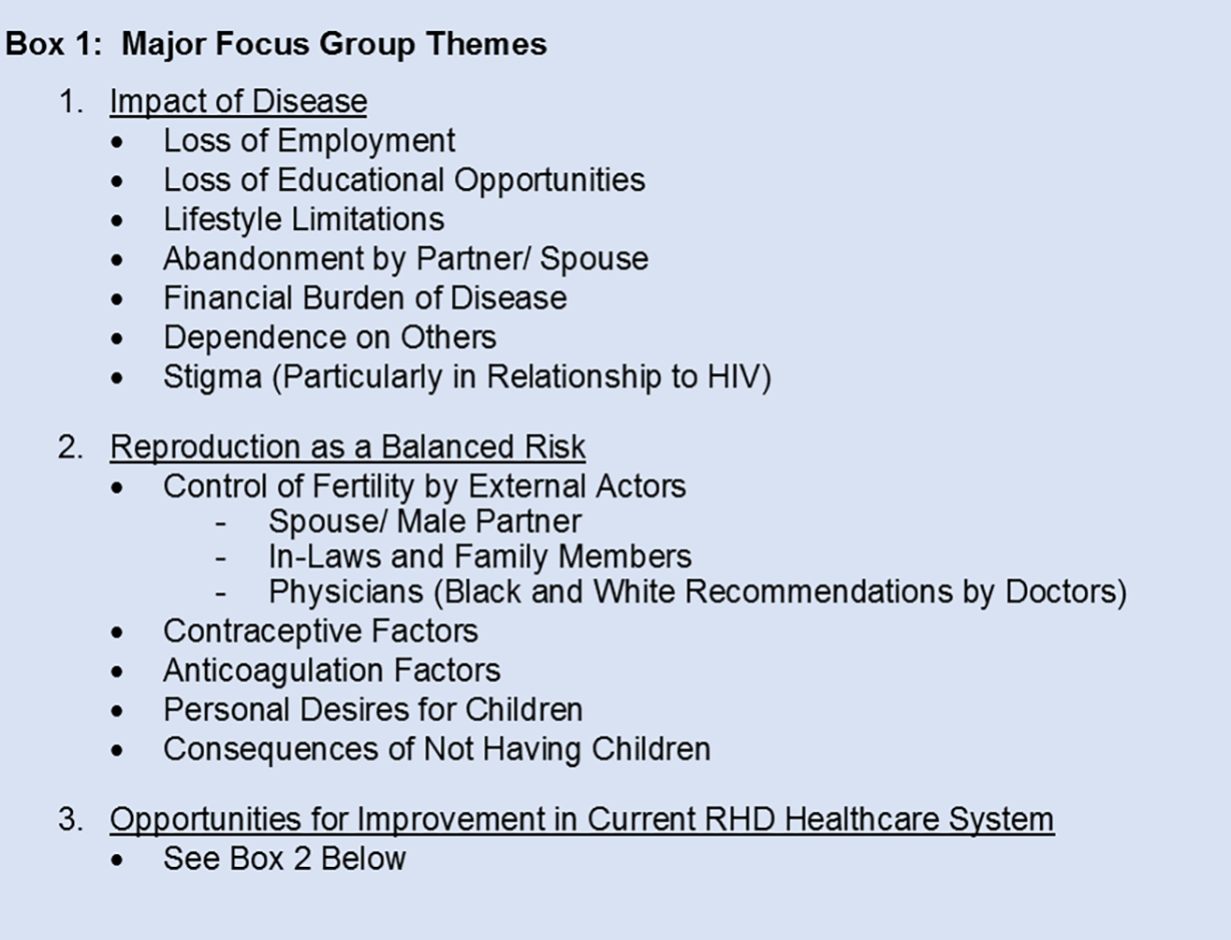
Focus group themes. List of the major themes and sub-themes encountered in the focus groups.

The focus group participants ranged from 22–59 (mean 35) years of age, with an average of two children. Most participants (68%) lived within 15km of UHI (Kampala, Uganda), and lived an average of 7 years with a diagnosis of RHD. 33% of the participants were unemployed, and 16% were university graduates (Table 3). Three major themes emerged from the focus group discussions, namely (1) the impact of RHD, (2) reproduction as a balanced risk, and (3) opportunities for improvement within the current RHD healthcare system (Fig 1).

**Table 3:** Sociodemographic characteristics of focus group participants (n = 25).

Variable	Range	Mean	Standard Deviation
Age (years old)	20–59	35	11.5
Number of Children	0–7	2.2	2.5
Number of Pregnancies	0–10	3.1	3.0
Distance of Home from Uganda Heart Institute	0–306 km	17 km	• 0–5 km: 5 • 6–10 km: 6 • 11–15 km: 6 • 16–20 km: 1 • 20–25 km: 1 • 26+ km: 6
Number of Years Since RHD Diagnosis	0–28	7	• 0–1: 4 • 2–5: 12 • 6–10: 3 • 11+: 6
Employment Status	• Employed: 33% (8/24) • Unemployed: 33% (8/24) • Student: 12.5% (3/24) • Homemaker: 20.8% (5/24)		
Educational Level	• Primary: 16% (4/25) • Secondary: 48% (12/25) • Vocational: 20% (5/25) • University: 16% (4/25)		
Religion	• Muslim: 5/24 • Adventist: 1/24 • Anglican/ Protestant: 8/24 • Born Again/ Pentecostal: 7/24 • Catholic: 2/24 • Jehovah’s Witness: 1/24		

#### Impact of RHD

Our participants reported several negative impacts from their disease (Fig 1). Those with significant symptoms had lost employment opportunities as well as educational prospects, often in the form of withdrawal from higher education. Nine of the 25 focus group subjects (36%) described some form of career/schooling limitation ranging from reduction in work hours to loss of jobs. Related, they also noted significant financial burdens of RHD. In addition to the high cost of medications, they also cited the expense of valve replacement surgery. One unexpected source of financial difficulty was that of infant formula, which a focus group discussant reported needing to buy because her diuretic medications limited her lactation volume. The formula cost her 250,000 Ugandan Shillings (approximately $70 USD) a month. These burdens often left participants dependent on others, including spouses, boyfriends, in-laws, parents, friends, and co-workers.

A subtheme frequently related was also abandonment or the threat of abandonment by male partners. Seven of the 25 focus group participants (28%) reported that they had been left by their spouses or boyfriends due to perceived fertility limitations, and nine (36%) reported fear of abandonment. This phenomenon was not limited to subjects who had been unable to conceive. A striking example was a subject who was abandoned by her entire family (including in-laws) and lost custody of her two children after being diagnosed with RHD. She was told by her husband “that he cannot stay with a woman who cannot give birth.”

Another subtheme that emerged was the considerable stigma experienced by patients with RHD. Subjects often compared their heart disease to HIV/AIDS. Three participants in separate focus groups reported being accused of having HIV instead of heart disease when they were seen taking their medications. In all, five of the 25 participants (25%) had been stigmatized in terms referencing HIV/AIDS. One focus group member reported that her classmates would even discuss whether they would rather have HIV or a cardiac condition. A consideration that was brought up in this debate was that “when you have a heart problem, you cannot give birth.” Another respondent in a different focus group supported this assertion independently, stating “when one gets HIV, they get you something such that you are able to give birth to a healthy baby” (referring to HIV Prevention of Mother to Child Transmission {PMTCT} measures). The stigma couched in reproductive terms was also seen independent of the HIV comparison. One participant was told “You are not supposed to get married because of your RHD. No man can handle you. You won’t even give birth.”

#### Reproduction as a balanced risk

**Fig 2:**
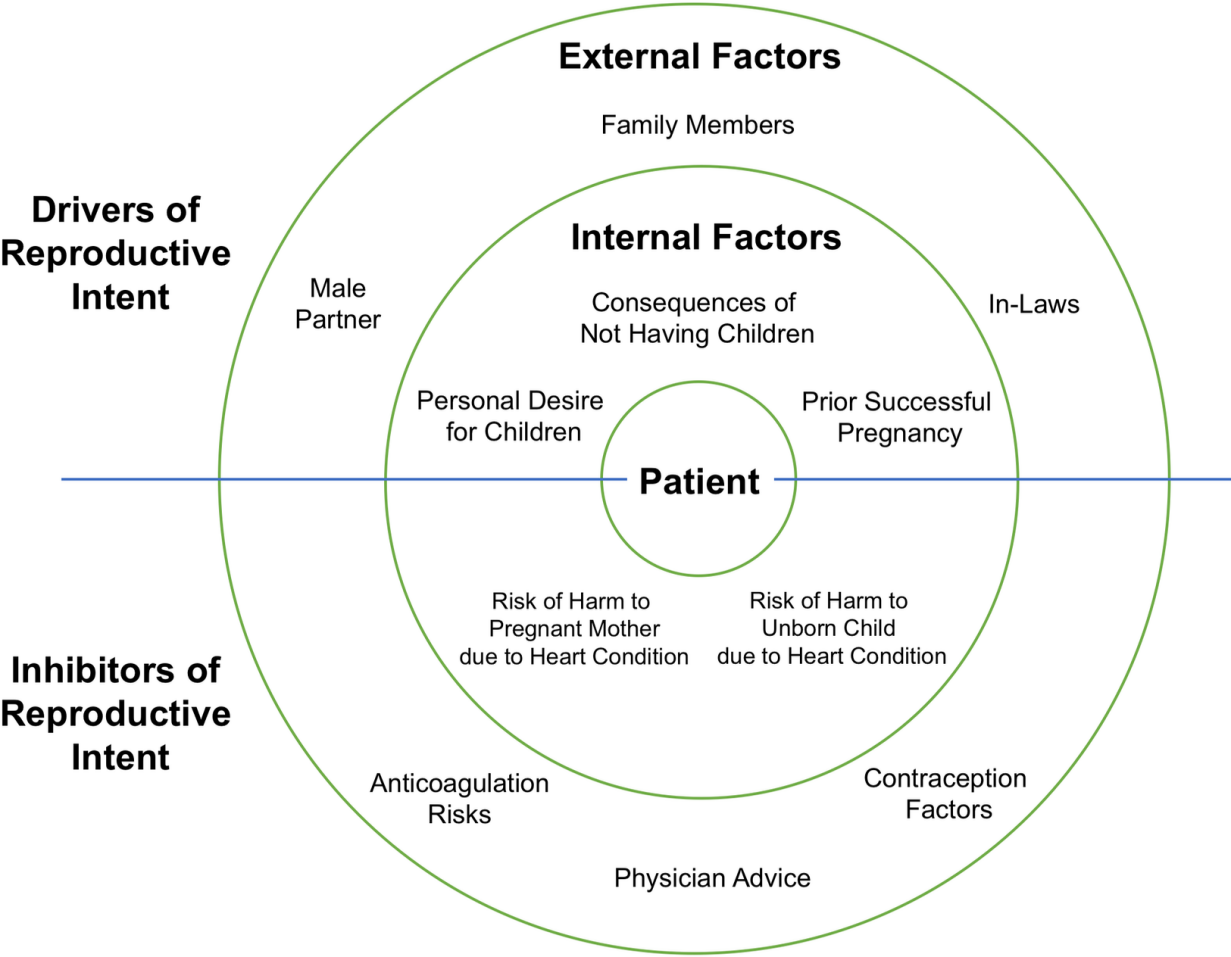
Reproduction as a balanced risk. Schematic depicting focus group theme of external and internal factors driving or inhibiting reproductive intent in women of childbearing age in Uganda.

Another major theme that emerged was that of childbirth as a balanced risk by patients with RHD, involving a number of drivers and inhibitors of reproductive intent (Fig 2). These factors could further be divided into external and internal forces.

**External forces:** Outside decision makers: Focus group participants revealed that reproduction is not the decision of a woman alone, and was influenced by other stakeholders. First, male partners were usually strong drivers of reproductive intent, both directly (by petitioning their spouses for children) or indirectly (due to women’s fears of abandonment if unable to bear children). One participant reported that the number of children she hoped to have “was to depend on how my husband was to treat me”. Other decision-making entities that drove childbearing goals included blood relatives and in-laws. On the other hand, respondents frequently described physicians and healthcare workers as the primary outside decision-makers inhibiting reproductive intent. Doctors were often reported as giving black and white (absolute) advice regarding reproduction, usually discouraging childbearing. Eleven of the 25 focus group participants (44%) recalled being told by their physicians that they cannot or should not get pregnant because of their heart conditions. One woman was told by a physician “that if I got pregnant…either myself or the baby, one has to die during delivery.”

Contraception factors: Many participants in the focus groups reported that given the recommendations that they had received from healthcare practitioners, they chose to be on a variety of contraceptive measures including oral contraceptive pills (OCPs), intrauterine devices (IUDs), emergency contraceptives, withdrawal techniques, and abstinence from sex. A subtheme observed was the presence of several misconceptions about the cardiac side-effects of OCPs and IUDs, including IUDs causing endocarditis or blood clots and OCPs causing hypertension, excessive bleeding, or thrombosis (Table 4).

**Table 4:** Focus group themes–selected exemplar quotations.

Themes	Exemplar Quotations
**Impact of Disease** ** • Lossof Employment** • **Loss of Education** • **Lifestyle Limitations** • **Abandonment by Partner/Spouse** • **Financial Burden of Disease** • **Dependence on Others**	“There was a time I had an introduction ceremony but my man refused to appear because his mother had told him, ‘You cannot marry that woman who cannot give birth.’” “The other thing they say is, ‘Will you handle that one? She is so costly and if you don’t have money you won’t handle her. It’s because she is costly; her delivery will require money and everything about her requires money.’”
**Stigma** **(In relationship to HIV)**	“I was in high school in the boarding section but I would take tablets every day and people would see me taking them. So they would say that it is HIV and that I was just hiding it from them.” “Can’t there be any other medicine they can get you for that time? For instance when one gets HIV, they get you something such that you are able to give birth to a healthy baby.”
**Personal Desire for Pregnancy**	“I separated with this guy, [but] my kid gives me hope. Sometimes I can be on bed feeling pain and then my kid comes and asks me ‘mummy, are you sick?’ Then I feel like a person cares.” “We should not be judged because we are also human beings. Much as we have this in our body, we have desires, we want children and we want families.”
**Reproduction as a Balanced Risk by Patients**	“If birth control can prevent me from getting pregnant, I will take it, because I know that I am not supposed to get pregnant when I am on warfarin because it is dangerous.” “[The doctor] told me that if I got pregnant yet I have a heart disease, either myself or the baby, one has to die during delivery”
**Control of Fertility by External Actors**	“My husband was okay before I got pregnant but when I delivered the second baby, he said, ‘now you will deliver even the third one.’ So I think he thinks they were just lies in the beginning now that I had delivered the second baby. So now he is like, ‘now you will have the third one and we end at that’ which is not easy since it is a risk you have to take.”
**Black and White/ Absolute Recommendations from Healthcare Providers**	“Whenever I ask my doctor about the right time I will get pregnant he just tells me, “you hold on I will tell you when the right time comes.” “Then he [the doctor] was like, 'Oh my God! You should not have any other baby.' So I wondered, 'Will this man [her husband] stay with me? Is he going to leave me?’”
**Misconceptions about Medications**	“I was using IUD but… it causes a risk of endocarditis. I couldn’t go for hormonal because of the risk of coagulation.” “Since my blood pressure had risen, they told me that if I added contraceptives it would further increase.” “They always tell me that the tablets that I swallow every day will cause me fibroids.”

Anticoagulation factors: Several focus group respondents volunteered awareness that warfarin could be harmful to an unborn fetus. All eight participants in one focus group remembered having been informed that warfarin was teratogenic by a healthcare provider. Some recalled that their physicians had told them to take contraceptive therapies while on anticoagulation as well. They also described awareness of the consequences of not being on anticoagulation if needed, including the risk of stroke and paralysis. There were also myths about reproductive side-effects of warfarin anticoagulation. For example, three subjects in two separate focus groups reported being told that warfarin therapy would cause them to develop uterine fibroids (Table 4).

**Internal forces:** Patients described multiple internal drivers and inhibitors of reproductive intent (Table 4). Most notable drivers were personal desire for children, reported by the majority of focus group participants. Asked to clarify, participants cited social expectations of fertility and fear of abandonment by spouses, friends, and family as reasons they wanted to have children. As for inhibitors, subjects often conveyed the understanding that heart disease posed hazards of mortality to both the pregnant mother and fetus. The desire for children was often expressed in terms of a risk, exemplified by one discussant who stated “I will get pregnant and I will endure each and every thing… I am ready to take the risk.”

Another subtheme identified was that of prior successful fertility encouraging future pregnancy decisions. Three women reported that they or their male partners either planned to become or became pregnant due to experiences with previous successful pregnancies. In one case, a participant already had two children, and though she was advised to stop having children, lobbied her husband to have a third. These respondents further implied that these experiences made them and their partners question the advice of physicians regarding the reproductive implications of their RHD diagnoses.

#### Opportunities for improvement in the RHD healthcare system

**Fig 3:**
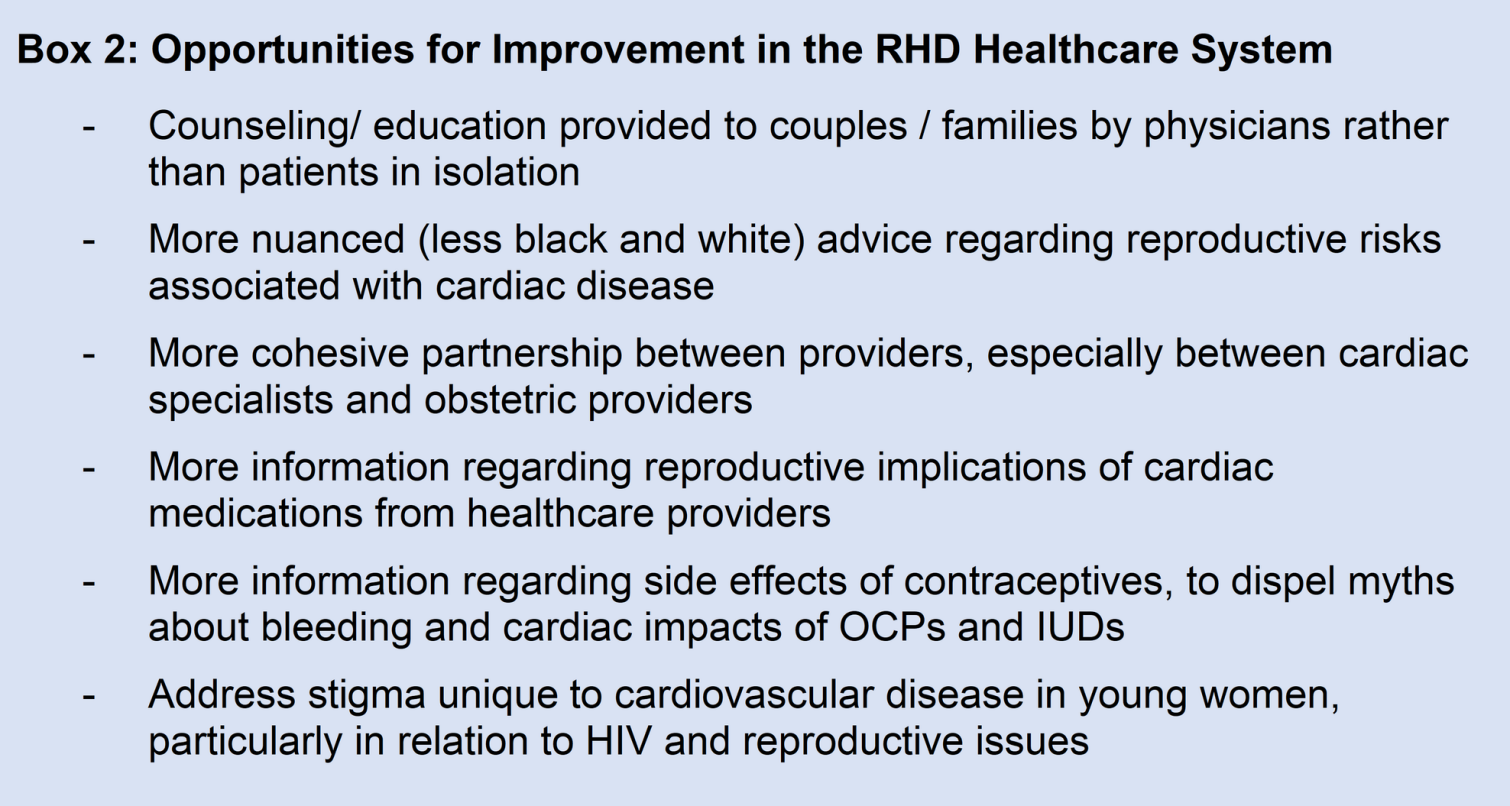
Opportunities for improvement. List of sub-themes encountered in the focus groups detailing opportunities for improvement in the current RHD healthcare system.

The third major theme described in the focus groups was that of potential improvements to the RHD healthcare system (Fig 3). Participants often recalled that their cardiac disease was explained to them without mention of the reproductive implications of the condition or its treatments. In other cases, they received contradictory or incorrect advice from both doctors and community health workers. Five respondents stated that physicians should involve male partners or family members in the discussion of the disease. One subject reported that her husband never took her condition seriously until her physician brought him into the office and explained her disease to him. She noted that “if [a] man is sat down and spoken to by health workers, there is a better impact than when [the patient] tell[s] him”.

## Discussion

To our knowledge, our study is the first qualitative analysis of the attitudes of women of childbearing age with RHD (in both developing and developed nations) regarding the impact of their illness on reproductive issues. As such, it contributes to a yet unexplored field of the experience of women living with chronic NCDs in poor countries. Our questionnaire findings corroborate existing literature on RHD patients, including low rates of birth control usage and the prevalence of women continuing warfarin while pregnant [16]. 72% of our survey respondents reported that being on warfarin would not reduce their desire to have children. Focus group participants also expressed belief in several misconceptions about the impact of contraceptive drugs and devices, which may explain some of the discrepancies between need and uptake. Myths about side effects have been reported as barriers to family planning utilization in Sub-Saharan Africa, including the belief that contraceptives cause fibroids [24–26]. Our findings, however, also showed a degree of sophistication in RHD patient concerns about contraceptives incorporating fears of cardiac or hematologic side effects such as bleeding, thrombosis, endocarditis, or hypertension. Furthermore, prior qualitative research suggests that family planning in rural LMIC settings is highly stigmatized and leads to poor adoption, even when resources are conveniently and freely available [25,27]. In addition, all questionnaire participants had been told by a physician that their hearts were not strong enough to support a pregnancy. This information, however, did not eliminate respondents’ wishes for children, as 62% reported that their RHD diagnosis would not affect their reproductive aspirations.

Regarding our focus group findings, many of the reported impacts of RHD were expected, including loss of economic/educational potential and financial burden of the disease. An unexpected source of financial hardship, however, was baby formula from stunted breastmilk production. A surprising number of women reported both fear of abandonment and actual abandonment by male partners, even after children had been borne. As for the theme of stigmatization, we were surprised to have encountered the frequent, spontaneous comparisons between cardiovascular disorders and HIV/AIDS in focus group discussions, with heart disease being rated more unfavorable compared to HIV. The self-management aspects of noncommunicable chronic diseases described by focus group participants, such as long-term medication dependence and lengthy duration of symptoms, likely inspired this association. Interestingly, reproductive potential was often cited as a reason why having HIV would be preferable to having RHD: participants and their peers described HIV PMTCT measures as making safe childbearing possible, while risks to mother and fetus from maternal cardiac conditions made having children risky or impossible. These novel results suggest the need for directed destigmatization initiatives targeting NCDs.

Data from the focus groups also illustrated the complex decision-making process female RHD patients undergo. It appeared that the women were generally aware that their heart condition posed a hazard to both the health of a potential mother and unborn child, and they used language such as “gambling” and “risk taking” to express this concept. The notion has been previously explored in qualitative research of HIV-positive women and women with renal failure, who recognize the potential for harm to children from their diseases or medications, yet still desired reproduction [28–30]. Drivers of reproductive intent characterized in these studies included the sentiment that motherhood provides esteem, value, and hope in the face of incurable disease [28–30]. The role of social expectations, women’s duty to bear children, and gaining societal value by becoming a mother were also cited. Women desire control over the reproductive process, however [24–26,31]. In the face of knowledge of this deeply personal risk, the degree to which male partners drove reproductive plans for RHD patients was striking. Social norms described in Uganda and neighboring Rwanda and South Sudan suggest that men control fertility decisions—even regarding contraceptive use [25,26,31].

Another driver of reproductive intent we characterized was the experience of prior successful pregnancies often convincing patients and their spouses that further fertility was possible, even counter to the advice of physicians. This type of decision-making based on anchoring to previous experience is corroborated by mixed methods investigation of other diseases such as in maternal choice to vaccinate children dependent on good or bad prior vaccination events and acute myocardial infarction (MI) patients delaying medical care based on comparison of their acute symptoms with prior MI symptoms [32,33].

Finally, our study participants proposed a number of ways that the current RHD healthcare system could be improved (Fig 3). First, subjects requested that cardiac healthcare providers weigh in on reproductive implications of cardiac illnesses. Similar sentiments have been expressed by young women with cancer, who noted that their oncologists often ignored issues of fertility during disease counseling [34]. Second, given the pressure placed on patients to reproduce by their partners, several respondents wished for more couples counseling opportunities by physicians. Third, the black and white recommendations provided by doctors left women disheartened or distrustful (especially if patients had prior successful pregnancies) and potentially less likely to heed their advice. In a meta-analysis of pregnancy in women with chronic kidney disease, subjects described warnings against pregnancy by doctors (expressed in terms of risk of harm or death to mother or child) as “overbearing” and “traumatic” [30]. Individual risk tolerance is an important variable when women are making pregnancy decisions, and should be taken into account by healthcare providers. Lastly, our focus group participants volunteered a number of misconceptions about both the cardiac side-effects of contraceptives and reproductive side-effects of warfarin. This points toward a need for providers to explain the relationship between cardiac and reproductive therapies together.

## Conclusions

In sum, our findings suggest that health programs targeting RHD in LMICs must pay special attention to female patients of childbearing age. Their disease puts them at increased risk for obstetric complications, a problem exacerbated by lack of women’s control over fertility decisions and misconceptions surrounding contraceptive measures. To this end, a multidisciplinary approach to heart disease and reproductive health is needed, to ensure both prevention of high-risk pregnancies and closely controlled deliveries. There may also be opportunities for patient education programs and community engagement, leading to better outcomes and patient empowerment. Additionally, the healthcare system should address stigma unique to cardiovascular disease in young women, particularly in relation to HIV and reproductive issues. Further research is needed to understand the characteristics and motivations of this population in order to better serve their needs in a manner that is both medically efficacious and also culturally sensitive.

## Supporting information

S1 Checklist COREQ checklist. Completed Consolidated Criteria for Reporting Qualitative Research (COREQ) Checklist for this research study.
(DOCX)

S1 Protocol Focus group interview guide. Interview guide used for focus groups.
(DOCX)

S1 Appendix Questionnaire consent form. (DOCX)

S2 Appendix Focus group consent form. (DOCX)

S3 Appendix Focus group transcripts as coded comments. (DOCX)
