# Supplementary material for: Motivations of women in Uganda living with rheumatic heart disease: A mixed methods study of experiences in stigma, childbearing, anticoagulation, and contraception
Source: PLoS One. 2018 Mar 28;13(3):e0194030. doi: 10.1371/journal.pone.0194030 (PMC5874006; doi:10.1371/journal.pone.0194030)
Supplement: S1 Checklist — Completed Consolidated Criteria for Reporting Qualitative Research (COREQ) Checklist for this research study. (DOCX) [file pone.0194030.s001.docx]

**S2:Checklist**

The below checklist was reproduced from Tong *et al.* *International Journal for Quality in Health Care*; (2007) 19;6:349-357 per *PLOS One* submission guidelines.

**Domain 1: Research Team and Flexibility**

*Personal Characteristics*

1. Interviewer/Facilitator – Focus groups were led by author H.N. Author A.W. also observed the first focus group and helped coach H.N. after the sessions based on the discussion.
2. Credentials – Author A.C.: *MD*; J.N.: *MBBS*; H.N.: *Social Worker*; E.O: *MBBS*, *PhD*; I.S.: *MBBS*; C.L.: *MD*; A.W.: *RN, PhD.*
3. Occupations:

- Andrew Chang: Clinical Instructor, Department of Medicine, Stanford University
- Juliet Nabaale: Fellow Physician, Uganda Heart Institute, Mulago Hospital
- Haddy Nalubwama: Social Worker, School of Public Health, Makerere University
- Emmy Okello: Professor, Uganda Heart Institute
- Isaac Ssinabulya: Fellow Physician, Uganda Heart Institute
- Christopher Longenecker: Assistant Professor of Medicine, University Hospitals Harrington Heart & Vascular Institute, Case Western Reserve University School of Medicine
- Allison Webel: Assistant Professor, Frances Payne Bolton School of Nursing, Case Western Reserve University

1. Gender – By author: A.C.: Male, J.N.: Female, H.N.: Female, E.O.: Male, I.S.: Male, C.L.: Male, A.W.: Female
2. Experience and Training: All authors are trained as medical professionals and clinicians and have taken part and published peer-reviewed primary literature in the biomedical sciences.

*Relationship with Participants*

1. Relationship Established – Focus group participants were not known to the investigators prior to the study. All participants had the opportunity to ask questions and express concerns during the consent-signing process.
2. Participant Knowledge of the Interviewer – The motivations and background of the study team members were made clear to the participants in the consent form as well as the focus group guide (see Supporting Information 1)
3. Interviewer Characteristics – The motivations and background of the study team members were made clear to the participants in the consent form as well as the focus group guide (see Supporting Information 1)

**Domain 2: Study Design**

*Theoretical Framework*

1. Methodological Orientation and Theory – As described in the manuscript, qualitative description methodology was utilized.

*Participant Selection*

1. Sampling – Consecutive sampling was utilized.
2. Method of Approach – Participants were selected from an existing registry of patients with rheumatic heart disease in Uganda.
3. Sample Size – 25 subjects were divided into three focus groups of 8-9 subjects.
4. Non-Participation – There were no drop-outs among selected subjects.

*Setting*

1. Setting of Data Collection – The focus groups were conducted in a secure, private room on Mulago Hospital grounds in Kampala, Uganda.
2. Presence of Non-Participants – Author H.N. served as facilitator of all three focus groups. As noted above, author A.W. was also present during the first focus group to observe the interactions and ensure appropriate acquisition of data.
3. Description of Sample – Please see Table 3.

*Data Collection*

1. Interview Guide – Please see Supporting Information 1.
2. Repeat Interviews – No repeat interviews were required.
3. Audio/Visual Recording – Data was collected via audio recording.
4. Field Notes – Field notes were made after the focus groups.
5. Duration – Each interview lasted approximately 90 minutes
6. Data Saturation – Data saturation was achieved via iterative analysis and coding of transcripts.
7. Transcripts Returned – Given subject distance from research site, remoteness, and variation in literacy/English proficiency, transcripts were unable to be returned to participants for comments.

**Domain 3: Analysis and Findings**

*Data Analysis*

1. Number of Data Coders – The data was coded by authors A.C., A.W., and J.N.
2. Description of the Coding Tree – Please see Figure 1 for the coding hierarchy.
3. Derivation of Themes – Themes were derived from the data rather than in advance.
4. Software – Data was managed using Microsoft Word, Microsoft Excel, and QDA Miner software.
5. Participant Checking – As noted above, given the subjects’ distance from research site, remoteness, and variation in literacy/English proficiency, the findings were unable to be presented to the participants for commentary.

*Reporting*

1. Quotations Presented – Quotations were presented in the manuscript and in the tables to illustrate each theme.
2. Data and Findings Consistent – There was consistency between the data presented and the findings.
3. Clarity of Major Themes – Major themes are presented in Figures 1-3 and Table 4, as well as in the body of the manuscript.
4. Clarity of Minor Themes – Minor themes are presented in Figures 1-3 and Table 4, as well as in the body of the manuscript.
