# Supplementary material for: Motivations of women in Uganda living with rheumatic heart disease: A mixed methods study of experiences in stigma, childbearing, anticoagulation, and contraception
Source: PLoS One. 2018 Mar 28;13(3):e0194030. doi: 10.1371/journal.pone.0194030 (PMC5874006; doi:10.1371/journal.pone.0194030)
Supplement: S1 Protocol — Interview guide used for focus groups. (DOCX) [file pone.0194030.s002.docx]

**S1 Protocol**

**Focus Group Interview Guide**

**Characteristics and Motivations of Women of Reproductive Age in Uganda with Rheumatic Heart Disease: A Mixed Methods Study**

**Introductory remarks: To be given by the moderator**

Thank you for joining us today for this focus group. We are here today to learn from you how people living with heart disease feel about certain medications used to treat rheumatic heart disease. In particular, we are interested in your perspective as women of childbearing age, since the Uganda Heart Institute is planning to start a women’s heart center to meet the needs of women with cardiac conditions. We have been studying data from a countrywide survey for the past several years, and wish to supplement this by having discussions with patients and community members to better serve your health needs.

You have been invited to participate in our study as a member of a “focus group”, a collection of patients of similar background. The format is not a group interview, but rather an opportunity for you to share your opinions and experiences on the topics we discuss today, ultimately allowing us to build a better health program.

There are several important rules for this meeting:

1. There are no right or wrong answers. We simply wish to hear your opinions on the matters we are to discuss.
2. All of the opinions and statements from today’s discussion must remain confidential and should not be repeated outside of today’s focus group.
3. Please do not discuss this meeting with people who were not present here at the meeting.
4. For your protection and to preserve anonymity, please choose a fake name which cannot be traced back to you. Every time you speak into the digital audio recorder, please state that “it is [your chosen fake name]” so that we know who is speaking when we review the record later.
5. The study team is the only group of people who will have access to the tape. Nobody outside this room will be able to trace your comments back to you.
6. Please speak one-by-one. Do not speak when someone else is talking. Also, speak clearly and loudly so the recorder can pick up what is said.

As the moderator, I am here to ask questions and listen, but I will not be participating in the discussion. Please feel free to discuss each question with one another. My role is to guide the discussion to collect the information most useful for the researchers.

My role, however, may mean that I will do certain things, including:

1. I may ask some participants who are more quiet to volunteer their thoughts, and others who have many great ideas to space out your comments so everybody gets a chance to share their opinions.
2. Some topics of discussion may evoke strong emotions from participants. We ask that you respect the feelings of all members of the group here. Again, please protect the confidentiality of all speakers today regarding these subjects.
3. If the discussion begins to stray off the subject, I may ask that we re-direct and re-focus our conversation back to the study questions at hand.

**Discussion Questions (For Patients):**

**General Questions:**

1. Tell me what you know about your cardiovascular illness (rheumatic heart disease)—

Prompts: What caused it, how it is treated, and how does it impact your everyday life?

**General Fertility Questions:**

1. What has your doctor told you about your heart disease and pregnancy? Has a doctor ever told you that your heart is not strong enough to support a pregnancy?
   1. How has this information impacted your desire to have children, if at all?
   2. Have you ever been offered birth control methods such as condoms, medications, or an IUD (intrauterine device)?
   3. How has the information about the strength of your heart in regards to supporting a pregnancy impacted your decision to be on birth control?
   4. Do you think there is prejudice or judgment directed against women of childbearing age who have heart disease and may not have hearts strong enough to survive a pregnancy?
      - If so, from who? – Parents? Husbands? In-laws? Friends/Neighbors? Doctors/ Healthcare providers?
   5. Are there barriers for women with heart disease to obtaining birth control therapies?
      - If so, what are the barriers?

- Cost of medicine/ treatments?

- Cost of travel?

- Distance from health centers/ pharmacies?

- Fear of medications?

- Judgment by family/ friends?

- Patients not interested in birth control?

**Anticoagulation Questions:**

People with rheumatic heart disease sometimes need to take special medications to “thin the blood”—in other words, make the blood less likely to form clots. Reasons to take these medications include having a heart valve replacement surgery or having an irregular heart rhythm. Some of you may be taking blood thinning medications now. Others might not need them now, but may need to take them in the future. All medical treatments have benefits, but they also have risks. Deciding whether or not to take the medication requires you and your doctors to weigh the risks and the benefits.

1. Has a doctor ever told you that you should be on a blood thinning medication like warfarin?
   1. If so, did she/he explain why it was important to be on a blood thinner? (in other words, the risks of not taking this medication)
   2. What are the benefits of taking warfarin if it is needed?
   3. What is a stroke? Do you know anyone who has suffered a stroke? Do you worry that you might suffer a stroke?
2. Has a doctor ever told you the side effects of taking a blood thinning medication like warfarin?
   1. If so, what side effects were described?
   2. Did she/he explain that being on warfarin could harm an unborn child during pregnancy or put a pregnant woman at risk of a significant bleed during childbirth?
   3. How has this information impacted your desire to have children, if at all?
   4. Were you offered birth control methods such as condoms, medications, or an IUD (intrauterine device)?
   5. How has the information about the side effects of warfarin impacted your decision to be on birth control?
   6. Do you think there is prejudice or judgment directed against women of childbearing age who have heart disease and need to be on medications like warfarin?
3. If you were told that you needed a blood thinning medication in the future to prevent a stroke:
   1. Would the knowledge that warfarin can cause harm to an unborn child affect your willingness to take the medication?
   2. Would you be willing to use birth control while taking warfarin? If so, what types of birth control would be most acceptable to you?
   3. Would the number of children you already have affect this decision to use birth control?

**Concluding Question:**

1. Are there any other thoughts or concerns you’d like to share with the study coordinators?
