# Supplementary material for: Motivations of women in Uganda living with rheumatic heart disease: A mixed methods study of experiences in stigma, childbearing, anticoagulation, and contraception
Source: PLoS One. 2018 Mar 28;13(3):e0194030. doi: 10.1371/journal.pone.0194030 (PMC5874006; doi:10.1371/journal.pone.0194030)
Supplement: S1 Appendix — (DOCX) [file pone.0194030.s003.docx]

**S1 Appendix**

**Consent Form – Questionnaire**

**Characteristics and Motivations of Women of Reproductive Age in Uganda with Rheumatic Heart Disease: A Mixed Methods Study**

**Makerere University School of Health Sciences Research and Ethics Committee**

**Title of Proposed Study:** Characteristics and Motivations of Women of Reproductive Age in Uganda with Rheumatic Heart Disease: A Mixed Methods Study (Questionnaire)

**Principal Investigators:**

- Dr. Juliet Nabbaale, Cardiologist, Department of Internal Medicine, Uganda Heart Institute/ Makerere University School of Health Sciences; (+256) 772634292.
- Dr. Christopher Longenecker, Professor, Department of Medicine, Division of Cardiovascular Disease, Case Western Reserve University School of Medicine; (+1) 2168447890.

**Introduction/ Purpose:**

Thank you for your interest in our research. You were selected as a potential participant because you are a patient at Mulago Hospital. Please read this form carefully prior to agreeing to volunteer for the study, and please ask any questions you have before you sign it.

We would like for you to answer several questions in a survey about the characteristics and motivations of women of childbearing age in Uganda with rheumatic heart disease, in particular regarding their thoughts of how the disease and its treatments impact decisions on pregnancy and family planning. Prior to you making a decision on if you would like to participate in this study, we must provide you information on the purpose of our research, how it may help you, and if there are any potential risks or harms to you. We will also outline your expected role in the study.

You have absolutely no obligation to partake in this questionnaire, however, and at any time during the process if you feel that you would like to end your participation, you may do so without any consequences regarding your medical care at the Uganda Heart Institute.

If you do take part in the study, you will be provided information regarding any new findings from our research that may cause you to change your mind about continuing your involvement with the study.

**Study Procedures:**

The questionnaire will be administered at Mulago Hospital. Approximately fifty (50) women will be asked to participate. It will take between ten and thirty minutes and be overseen by a trained research social worker following clinic appointments. Patients who are illiterate will be able to respond to questions verbally with answers recorded by the research representative. The survey will be provided in English or Luganda.

The questionnaire will not collect any patient identifying information—that is, no personal details that may link your statements to your identity will be recorded. Nevertheless, all written materials will be kept confidential.

**Risks:**

Our study has a number of risks. First, since we will be asking you your opinion on sensitive subjects such as health, pregnancy, and childbirth, you may feel awkward or uncomfortable. You are welcome to skip any part of the questionnaire that makes you feel awkward or uncomfortable. Second, though we will not be collecting any patient identifying information, there is always the risk that there may be a breach of confidentiality following these studies.

**Benefits:**

There may not be any direct benefits to you for taking part in the project, but the information gained from your answers and comments will contribute significantly to our understanding of heart disease. This information will be used to create programs which can better identify and serve the needs of women of childbearing age with rheumatic heart disease in Uganda.

**Alternatives to Study Participation:**

You may choose to not partake in this research at any time. There will be absolutely no consequences for your health care based on your decision to either stop participating during the study or never participate in it at all. If at any point you feel uncomfortable during the study, you may also choose not to take part in that part of the questionnaire or study.

**Financial Information:**

There is no cost to you for being involved in this study.

**Confidentiality:**

All data gathered from this study, including your questionnaire answers and your verbal comments (recorded or not), will be kept confidential and private. All records will be kept in a locked file accessible only to study coordinators, the researchers, the institutional review board (which exists to protect the rights of participants), and regulatory groups. Any electronic transcriptions of the data will be protected with digital passwords known only by the research team. All patient-identifying information will be removed from the data. All identifying information such as your name will be replaced in our study files with numbers and codes for written transcriptions. Records will be kept private by the full extent of the local law.

**Privacy of Protected Health Information:**

By consenting to this study, you authorize researchers from Mulago Hospital, Makerere University School of Health Sciences, Case Western Reserve University, and Stanford University to access, use, and disclose information about you, your medical history, and any other data gathered from the survey for the purpose of better understanding the characteristics and motivations of women of reproductive age with rheumatic heart disease.

This information may be used or disclosed to other individuals who are conducting or overseeing this study including the study sponsor, the Institutional Review Boards of participating institutions, and any US, European, Ugandan governmental, regulatory, and accrediting agencies. Note that laws in countries outside of Uganda regarding privacy, use, and disclosure of health information may be less comprehensive than those in Uganda. Once disclosed, your information may be redisclosed by others who are not required to maintain the privacy of your information. You are allowed to withdraw yourself from the study and withdraw collection of new data about yourself at any time during the study, but data already obtained may be continued to be used and disclosed. This authorization has no expiration date.

**Summary of your rights as a participant in a research study:**

Your decision to take part in our study is purely voluntary. As such, choosing not to participate will not affect your health care in any way. You will not be penalized in any way. If you do decide to participate, you may quit at any time for any reasons without impact to your health care and without penalty in any form. If information from this study is published or presented, your identity will be kept confidential. Any new information gained during the study that changes the risks/benefits of being involved in this study will be provided to you so you can choose to change your mind at any time.

**Disclosure of your study records:**

All efforts will be made to keep your identifying personal information from this study confidential, but absolute confidentiality cannot be guaranteed. This information may be accessed and reviewed by the Institutional Review Board.

**Contact Information:**

The interviewer has discussed the above information with me and offered to answer my questions. For any additional questions, I may contact Dr. Juliet Nabbaale tel: (+256) 772634292 or the Chairperson, School of Health Sciences Institutional Review Board (MakSHS-IRB) or Uganda National Council of Sciences and Technology. Tel: (+256) 020090376 / (+256) 772-404970 or (+256) 41-250431.

**STATEMENT OF CONSENT:**

_________________________ has explained to me the study process, risks, and benefits of the study. I am aware of my rights as a participant in this research and understand that my participation in the questionnaire will not affect my job, job security, or healthcare. Information regarding my identity will be concealed. I understand that I may withdraw from the study at any time. By signing this form, I do not waive any of my legal rights but simply note that I have been made aware of its process and am now volunteering to participate. A copy of this form will be provided to me.

| **X** |  |
| --- | --- |
|  | Signature of Participant Date |
| **X** |  |
|  | Printed Name of Participant |
| **X** |  |
|  | Signature of Witness to Participant Signature (If Participant is Illiterate) Date |
| **X** |  |
|  | Signature of Person Obtaining Informed Consent Date |
| **X** |  |
|  | Printed Name of Person Obtaining Informed Consent |
| **X** |  |
|  | Signature of Principal Investigator Date |
| **X** |  |
|  | Printed Name of Principal Investigator |
