# Supplementary material for: Motivations of women in Uganda living with rheumatic heart disease: A mixed methods study of experiences in stigma, childbearing, anticoagulation, and contraception
Source: PLoS One. 2018 Mar 28;13(3):e0194030. doi: 10.1371/journal.pone.0194030 (PMC5874006; doi:10.1371/journal.pone.0194030)
Supplement: S3 Appendix — (DOCX) [file pone.0194030.s005.docx]

**S3 Appendix: Coded Comments**

**Supplemental Material: Coded Comments from Focus Group Participants by Theme / Subtheme‡**

# Patient Understanding of RHD

*Interviewer: What do you know about RHD?*

***Respondent:*** RHD is a heart disease caused by an infection, streptococcal infection which can start in childhood but manifest in adulthood. So when the disease comes in your body, there’s like an immune response; the body tries to fight it and in the end, it fights itself and affects some of your heart structures.

*Interviewer: Well thank you so much. Now let’s look at the causes. I have heard something about it but in your opinion, what causes RHD? Respondent X, you have something?*

***Respondent:*** Yes, it’s from the tonsillitis

***Respondent:*** Yeah, so if you don’t treat them there is that bacteria which goes to the heart and starts damaging it.

*Interviewer: Well let’s begin; what do you know about the rheumatic heart disease?*

***Respondent:*** The doctor told me that I was not born with a heart disease but to get this illness, I used to get sores in the throat and that I didn’t swallow [take] modern medicines but local herbs like *“Omwetango”* (local herb) and although it would stop and I would feel no more pain in the throat, those infections went down. She said that the tissues in the throat are similar to those on the heart. So they started consuming them; she told me that they started eating up the doors (valves) of the heart and when they ate them it caused that problem. However in the beginning I didn’t know it since they couldn’t explain it to me. Whenever I felt the wounds I would go and buy some drugs for sore throat until when I grew up and was not eating *Omwetango* (local herb) anymore and still I hadn’t yet known the cause of the pain in my heart. However now the longer I lived with that pain, I learnt about it; whenever I get those sores I rush and also when I am given drugs, I swallow [take] my dosage and make sure that I complete it.

***Respondent:*** Personally I found out about it when I always had chest pain. I was also coughing a lot. I was coughing a lot until I reached an extent of getting tonsillitis. Now just like what that lady has said, *“Omwetango”* (local herb) is what I was using too.

When I went in for a scan they told me, *“You have a heart problem and you must go to Mulago.”* When I came here they told me, *“the heart is sick and the valves are blocked. The blood flows out and in and it doesn’t flow well in the body.”* So I found out that maybe it is those tonsillitis that cause the problem.

***Respondent:*** I also began with coughing; when I coughed for a long time I was taken to some hospitals and we also boiled mango leaves but it didn’t help. Then there was a certain doctor called X who used to stay in X but was working here. He said, *“Take her for a blood test because she might have contracted TB or a heart disease.”* Then one of my in-laws took me somewhere in X and when I was tested, they said that it wasn’t TB. Then X brought me here and they took some x-rays and then told me that my heart widened and formed a pool. They said that when the blood comes it rests together with the water and that’s what causes my coughing.

***Respondent:*** They told me that the cause of that illness is fire; cooking on fire. I spent a long time cooking on fire and they said that maybe the fire got into me. Now while I was still in X, it started as sores so I swallowed the sore tablet and then felt a rapid heartbeat here. Then they took me to the hospital and they treated me for a long time before figuring out that it was the heart. I went back home but then my condition worsened, so they took me back and gave me another type of drug and again before finding out that it was the heart. Now when they took me back when the illness couldn’t stop, I asked them to check me. Then they put me in a machine and checked me. They first feared to tell me but they asked me to spend weeks without eating or drinking. So they found out that it was the heart and then they told me, but I know that it is (caused by) the fire.

***Respondent:*** Cough; I frequently got a cough and whenever I got it I couldn’t have necessary medical attention to get treatment. So the infections accumulated and sloped down to the heart and ate the valves. So by the time they brought me here the blood would mix up.

***Respondent:*** It started with a headache and I would feel dizzy but whenever I delayed eating I would feel sores and pain inside me. I would feel like something burning my heart and whenever I talked to someone they would say that they were ulcers. So I got ulcers drugs but it still remained. Whenever I ate I would vomit and that’s how it went on; I would feel dizzy and that would make me faint.

***Respondent:*** I think it was caused by the severe headache I used to get.

*Interviewer: So please tell me what you know about this illness. What do you think caused it?*

***Respondent:*** I was told that in my childhood I might have had tonsillitis but didn’t get enough treatment. So the infection moved down and ate up the valve.

## Patient Misunderstandings about RHD

*Interviewer: Well thank you so much. Now let’s look at the causes. I have heard something about it but in your opinion, what causes RHD? Respondent X, you have something?*

***Respondent:*** According to my perception, one of the causes is that sometimes kids are born with a heart problem; heart complications. Recently I was talking to a lady at the reception and she was like, *“the kid has three holes!”* Another perception I have is that

the lifestyle we are living nowadays has turned into heart diseases because of the technology we are consuming, the processing. Yeah, that is it.

***Respondent:*** Well what causes it, like my colleague has said, it is that infection which starts with tonsillitis but I think it has some related risks like poor housing conditions. In fact, from when I got to know, in the morning I wanted all the air outside entering my house so that I don’t get this condition in [the] children. It can hide in the body and you get to know later, at the last hour when you get a serious complication. In fact, I remember one time I was treated for hypertension since I had trouble climbing the hill. So they diagnosed me with hypertension and put me on antihypertensive(s) which I later stopped until I got pregnant, delivered and then they told me that it was RHD.

*Interviewer: Well let’s begin; what do you know about the rheumatic heart disease?*

***Respondent:*** The doctors told me that I had worried too much and got stressed. Then I started feeling pain in the chest and would always feel rapid palpitations. There was one time when I fell down and I was brought here in Mulago. When it [the chest] was checked they told me that the vessels were blocked and the next time they told me that I got some infections; they said that I had a severe fever and then the heart was covered with pus and then the infections ate it.

***Respondent:*** Coughing a lot; I always cough. In fact it started from childhood and people would say that maybe I had TB, but they didn’t do much; they would just take me to clinics. So the thing worsened and worsened.

*Interviewer: So please tell me what you know about this illness. What do you think caused it?*

***Respondent:*** The doctor who checked me said that it was caused by an infection. I got that infection while I was still young. I would say I cannot give birth because whenever I get pregnant that infection worsens.

***Respondent:*** I think mine was caused by stress, because one time I was coming from school with my child after she had just been graduated from Top class to Primary one and then a truck carrying sacs of coffee passed by us. Then its tyre dropped off and hit my child and she died. So from then I would always get frightened

***Respondent:*** I also have that symptom; they told me that one valve is about to be blocked. So I also think that it was caused by stress because my man went abroad and it’s almost twelve years ever since. So that has got me so stressed [breaks into tears] Then one day I read messages on [cell phone texting application] from another woman whom I think he was trying to ask out but the man said that what she was saying wasn’t true. [Crying] so the moment I put the phone down, my heart beat so fast and from that time it started falling sick.

***Respondent:*** I think my heart started falling sick from the time I gave birth to my second born. That child was sickly and would get convulsions all the time and whenever she had them she would fall down and then I would start shaking and feel like my heart beating so fast. Now when the pregnancy was ready, I went for delivery but the doctor told me, *“Never get another pregnancy because your heart is sick and you might not be*

*able to push another child. If God blesses you and you deliver properly this time, please don’t get another pregnancy.”* Well I didn’t take it so serious after giving birth but it was after about 12 years that I started feeling rapid heartbeats all the time. Whenever I went to the hospital they would tell me that I am hypertensive. It was until the doctor whom I was seeing regularly said, *“It cannot be hypertension all the time”* because the rapid heartbeats would occur every time and then I would be admitted for about three days and leave. Then within one week, I would be admitted again until he suggested, “I think you should go to Mulago Hospital such that your heart is checked.” Then I came here and confirmed that I am a heart patient.

## Patient Understanding of Medications/ Treatments for RHD

*Interviewer: So what kind of medicine do you know that can...?*

***Respondent:*** According to me, it is not only treated by drugs but it also has conditions; there are certain foods we are not supposed to eat like foods containing vitamin K ***Respondent:*** To add more to what they have said, I do always come here for the INR which is called the International Normal Ratio. I do several, like every month I have to do it and I do take warfarin. I even take Benzathine Penicillin which is the injection I receive every month. That’s the treatment that I take.

***Respondent:*** I think it depends on the level when you are diagnosed, like if it is still early and that infection is just starting to affect your valves, I think if you are put on antibiotics you can recover and be free of the disease but if it has already affected your heart valves then you are treated at whatever level you are on. Some people may need surgery while others may not need. While others may need warfarin, others might not need it for example personally I have not been on warfarin since diagnosis and I have been stable.

*Interviewer: Do you know of any risk of not taking the drugs?*

***Respondent:*** It’s the same only that with the effects... Well warfarin is the one which controls the INR, so when it goes high, maybe if you didn’t take your tablets well or if you didn’t come for checkup, you may start bleeding from the body openings or sometimes you may receive a clot in the eye. Recently I got one myself and I wondered where it came from. I waited for like three days and it disappeared but when I told my doctor about it, she said that when you are on warfarin you should expect those slight effects although the INR was normal.

***Respondent:*** Even with the blood clotting, for instance when you cut yourself you can bleed for more than five minutes. He told me that when you cut yourself you hold on like for five minutes

*Interviewer: Has a doctor ever told you that you need warfarin to help to prevent blood clotting?*

***Respondent:*** She said that the blood would be in a good state where it is not thick or so watery. So she tells you how to apply it such that you are able to get an improvement ***Respondent:*** I was told that since I was inserted artificial valves, the (tiny) metals, it requires that the blood is not so thick but instead watery such that those metals can pump well and the blood doesn’t stick on the metal.

***Respondent:*** They told us that warfarin helps if the blood clots. It helps to loosen up the blood such that it flows well in the veins. Still if you delay taking your dose, the blood could become water, become too much and then starts coming out through the mouth, the nose and every part of your body. So you have to stick to the dose they have told you and also return on the dates you have been told such that they check your condition.

***Respondent:*** They told me that to swallow [take] those drugs, you have to always return; they give you a period to return. Although from where I was operated I was told to return every month, here they can give you a return date, for instance every three months, such that you come and they check the blood. If it is thick, they have a way they change the dose. If it is watery they still have a way they handle that. Here at Mulago I learnt something; Dr. X told us that there is a range in which one has to be – the normal range for someone who has valves is 1 – 3.

*Interviewer: What side effects of taking warfarin did the doctor tell you?*

***Respondent:*** I was told that if I get an accident or if I get a cut or a toothache, I shouldn’t go to any other hospital but instead come here directly. They told me that that is because here they know that I am on that medication but other doctors might not be responsive enough that I would run out of blood since the bleeding would be too much. That is the problem with it.

***Respondent:*** Over bleeding

***Respondent:*** The other thing about warfarin is that they stopped us from eating greens. Greens! In fact I cannot even eat food; I don’t eat. They say that if you are on warfarin you shouldn’t eat greens. So that issue of greens is so bad.

***Respondent:*** They barred me from eating beans, greens and cabbages. However by the time they stopped me from eating all those things, it was ten years after my operation. Otherwise in the beginning they told me to eat whatever I wanted. It was only after ten years

***Respondent:*** The doctor told me that if you are on warfarin and get pregnant, it could affect the unborn baby. That’s why he advises you that if you are preparing to get pregnant, well sometimes you can get an unplanned pregnancy but what you have to do first is to come to the doctor the moment you find out. He would have to first stop you for the first trimester- the first three months. He said that they would put you on injections and then monitor if they have to put you back (on warfarin) or keep you on injections.

*Interviewer: Now some people have to be on certain thinning medication to avoid clots in blood. Have you ever heard about such medicine?*

***Respondent:*** They told me. They told me that I swallow [take] it such that my blood would loosen up and not create clots because if it does so, it would stick in one position and then cause a problem.

***Respondent:*** They told us that if you don’t use it your blood could clot.

***Respondent:*** Like I was saying, when I left [business institution] there was a time when I was from church. While seated in a taxi I felt like I couldn’t get up. I went to the hospital and when I was tested the nurse found that it was a fever. When she noticed that it was a fever but I looked like a disabled child, she referred me to X. When I went to X they referred me to Imaging Center and they put me on a certain medicine called Neurontin. Otherwise I was told that it was kind of a stroke. I had it for so long, for years and years, until it relaxed gradually. Then they took me off the Neurontin and I remained with Aspirin. Then they told me that the chances are high if I stop taking Aspirin or that it is 99% possible to happen again. They said that I have to take it for life. So I didn’t know immediately that it was necessary to. Actually I first came here (later on) and then suspected that maybe it was (all because of) the heart disease and besides, they told me that the other clot disappeared. In fact they didn’t find it there. All they said was that it dissolved.

## Patient Misunderstandings about Medications/ Treatments for RHD

*Interviewer: So did they explain to you why it is important to be on those medications?*

***Respondent:*** They just told me that it will thin the blood but I don’t even know why. ***Respondent:*** My doctor told me that if I stop taking it the blood would be thick and I would get a stroke and move in a wheelchair. It’s so scary.

***Respondent:*** Yeah, it is stroke and also if I over take it, I am to get fibroids.

***Respondent:*** They didn’t tell us that but inside the warfarin (package) there is a note. If you read what’s written there, they indicate that if you are pregnant you shouldn’t swallow [take] that medicine. It could change your unborn baby into blood and then you start dripping blood. Consequently you get a miscarriage.

***Respondent:*** I was explained to the same way Respondent X was.

***Respondent:*** I was also explained to the same way.

***Respondent:*** Sometimes they tell us that if you take that medicine for so long sometimes the liver gets sick or the uterus. So that would make me discouraged. Personally I have an auntie who told me, *“Please don’t swallow [take] that. If you swallow it a lot it will result you into other types of illnesses.”*

***Respondent:*** They always tell me that the tablets that I swallow [take] every day will cause me fibroids.

***Respondent:*** Some of them tell us that you would rather pound the tablets into powder and then swallow [take] because whenever you swallow a full tablet, it would pile on another tablet and consequently it causes a fibroid or to spoil the liver.

*Interviewer: Which health workers tell you all that?* ***Respondent:*** Health workers in our communities *Interviewer: Is it health workers or other people?*

***Respondent:*** Sometimes it is the health workers. Or one could tell you, *“After swallowing the medicine you should drink a lot of water such that it dissolves when it gets inside.”*

*Interviewer: What hindrances to accessing family planning methods do your colleagues that have this illness tell you about?*

***Respondent:*** Some say that they make them sick. They say that they cause fibroids if one swallows [takes] tablets for instance.

# Impact of RHD

## Impact of RHD on Daily Living

*Interviewer: Now how has it impacted your daily life? How has your daily life been affected by RHD?*

***Respondent:*** For me at first I didn’t know that I had a heart disease. I could do each and everything. I completed my studies, I started working in [year] and I was okay. I had a job and I was happy but when I started falling sick in [three years later], then they told me that I am sick. So I used to work but I could not work anymore, I used to walk quickly but I started walking slowly and it reached a time when they had to lift me up. They had to do each and everything for me. I lost my job

***Respondent:*** Like if you were working but not working anymore, you are not enjoying, you used to go out but you can’t go out in cold days and every time you are sick and have to go to the hospital. But now I am okay.

***Respondent:*** you feel like your life is limited. In fact, even while revising, you lose hope and say, “I don’t think I will live for more than ten years; it may catch me from the middle.” Especially whenever you come here and see a dead body and that person is from the heart institute, you think, “I could be the next” and things like that are what cross your mind that time.

***Respondent:*** It has not affected me that much. Before I knew that I had it or before I got it I was the most hardworking girl at home; I used to do all the domestic work at home alone in a day. So when I got this disease, it still didn’t change me; I can still do everything alone because my sister is lazy. So I find myself doing everything. Of course I get tired but I find myself finishing everything. Maybe after doing them I go and rest for some few minutes but I get fine so quickly. So it hasn’t affected me that much apart from, there is this life you can’t enjoy that others do, like going to club because of that noise. The noise affects me so much that I feel like my heart is coming out

***Respondent:*** Well like they have said, sometimes you don’t live life to the fullest; you feel like you are limited not like others. Personally I even fear to sleep in the house alone. I make sure if I am sleeping in the house there is someone in and I make sure that I have airtime on my phone such that in case anything happens... Now with the daily work, it affects the way you do things at home. You would feel you don’t do like how you really want to do something. Even if it is house work, you feel like you are tired. If say you have are organizing the bedroom, you know that you won’t organize the next one because by the time you finish here you would have to stop much as you would want to organize the whole house. So you don’t have that energy. Also, even at home, there is this labeling that people put there; *“that one is sick”* or *“she has a problem”*.

Even our own family members do it; sometimes I hate it when my husband reminds me, *“don’t you know that you are sick?* “and then I would start denying, *“Am I sick? Do you see me in bed?”* So although you try to blow people’s comments away, still they come to you and you feel hurt. Sometimes I would want to eat something but then someone tells you, *“don’t eat that, don’t you know that you are sick? It will affect you.”* So you feel that you are limited somehow and you don’t do your work to the fullest. Sometimes I feel like I want to jump around with my sons but I couldn’t do that vigorous exercise that my son would want me to do with him. So you feel limited in other words. And even at work, you know you have to work with people who have all the energy but sometimes you feel that you are slower and your work is affected. Sometimes you feel that you have to sit down for some time. Someone might not even understand what you are really feeling; I remember one time I had to sit somewhere and tell people, *“You people I see things moving”* but you know someone cannot understand what you go through. Otherwise every day we get hope to move on, you have a positive mind and also decide not to think about the disease. I just forget it and do what I am supposed to do, take my medications and try to rub it out of my mind such that I can move forward.

***Respondent:*** Yeah, that’s it. I had no choice but to go for it. Now I can live but like she has said that she cannot club, me whenever I want *[others laugh]* it’s better to break the laws once in a while. So I go and club although it feels uncomfortable, to me I can say that this the life I want and I can spend there like an hour and then come back home. In fact, even my mum has this mentality of always reminding me *“...you know you are sick...”* that thing really pulls me down and it takes me back. Well I know I am sick and I don’t need to be reminded; it feels bad. Then everyone has their eyes on you, like you are on a look out; *“how are you?” “How are you feeling” [others laugh]* the care is too much although it’s okay.

***Respondent:*** That has to happen because you can study with colleagues and after school some of them get jobs, others marry or get married but you are still there! Now if you later on start communicating with each other on phone, they would say, *“What are you up to now? You are not married yet? What happened to you?”* and things like that. So you would feel... even if you try explaining to her/him. So you would still feel hurt and yet you too didn’t want it.

*Interviewer: Thank you. So how have your normal lives been affected or changed because of this illness?*

***Respondent:*** I have been affected because I am not able to do any serious work anymore compared to what I used to do. In fact even at my job they told me, *“according to how your illness is, we shall keep you. We won’t dismiss you such that you are able to continue taking care of your children.”*

***Respondent:*** Eating; you could get food and enjoy eating it but my problem is that even when I eat just a small portion, I would feel like my belly has swollen and it feels uncomfortable. The other disturbing thing is that I still have some pain even in the chest.

***Respondent:*** Now with my weight, people say, *“why don’t you do some exercises?”* but whenever I try to my joints would pain. So I don’t know what kind of exercises to do apart from the pushups while on bed and with a device to support me. Otherwise I cannot do the type where I have to jump.

## Impact of Disease on Reproductive Desires

*Interviewer: So how did that impact on your desire to have children?*

***Respondent:*** I was scared of course; I feel like I don’t want because when I hear of the impact of getting pregnant I feel like I like my life so much. I need a baby but no. Even me I can see that I am [age] and I can see that I am young but I ask myself... In fact even other people that have a similar problem like mine tell us that their doctors tell them that there is a certain age, that when you get to 30 years of age you would then be unable to [re]produce. So I start getting worried and just say me I have a have a brick eye and don’t look upon marriage.

***Respondent:*** Yeah. So that affected me and then I started being stressed. I started feeling stressed because of my family and children. In fact I do not even have any hopes of getting a man and then giving birth.

***Respondent:*** For some of us that haven’t yet given birth, whenever you hear about someone else who has four or five children but has that problem, you wonder in your heart, *“will I also give birth?”*

*Interviewer: So when you were told about the risk to the unborn baby when you are taking warfarin, how did that affect your desire to get pregnant?*

***Respondent:*** There is no way

***Respondent:*** There’s no problem

***Respondent:*** I think it makes no difference because if you get a problem you can see the doctors and they help you.

***Respondent:*** I had already given up with giving birth by the time I fell sick.

***Respondent:*** Of course you get affected. Now because I have two daughters, it affected me but you just have to live. Personally I didn’t know that I cannot... In fact sometimes I think that maybe I gave birth to these children when the heart is sick and while I was not yet on treatment, but how about now when I am on treatment!

## Impact of Disease on Pregnancy

***Respondent:*** For me I have suffered for so long. When you are pregnant you can’t do anything but just sit there. You get so tired very fast that you couldn’t even walk.

***Respondent:*** Going through the pregnancy was also hectic. At one time, they prescribed for me Spironolactone but I didn’t swallow [take] it. I was like, *“I can’t swallow [take] it because of the risks I have read about it”* I just continued with my Lasix and Atenolol and ate the potassium rich foods because they were telling me that Lasix is going to drain out my potassium. So I had to start medicating myself and even before the Spironolactone; I had to look for potassium rich foods and start eating them because I didn’t want to take the Spironolactone. Then through the pregnancy, I was okay through the first pregnancy but then towards 36 weeks, they told me that I had oligohydramnios; there was little fluid where the baby would sit and they were suspecting that it could be (due to) the Lasix because they told me, *“now there are reduced fetal movements and the baby is going to die in your stomach because he is not playing since there isn’t any water in which he can play.”* So I was taken for surgery there and then. Imagine I had come for duty with my bag ready for work but the next hour they are taking me to the theatre with completely nothing but just because of that. I think it w/as because of the Lasix that I was taking. So I went to [the operating] theater and delivered but after pregnancy, I get this pulmonary edema and I was coughing throughout. I was just watching my baby there helpless, he couldn’t even breastfeed. I had to start my baby on formula milk as early as two days. Then after that I couldn’t breastfeed all through since I didn’t have enough breast milk because Lasix was limiting my lactation. I had to put my baby on formula milk and that was an average of

250,000Uhs a month since my baby was taking a lot. That is the budget we have been spending. So the whole process was not easy but I went through it.

***Respondent:*** Well they told me. After getting pregnant, I was in a very bad condition so they took me in for an abortion and by that time we were still at Ward X. So I was taken there and they performed the abortion. When they brought me in to cut my tubes, my condition worsened so they gave up.

## Loss of Employment / Educational Opportunities

***Respondent:*** Yeah. Personally, this RHD has given me a hard time because I was at school, at [University] but this semester I didn’t go back to school; I had to apply for a dead year because whenever I move I get so tired fast. Actually, even right now, I was from down but I feel like I was dying. Even from home, I don’t do work because when I bend I get so tired. Actually, at the moment I am not able to bathe myself; they just bathe me. So it has already done a lot of bad things to me especially to my education. Actually, I applied for a dead year and maybe if I become fine I will resume studying next year.

***Respondent:*** Yeah. Before my surgery I wasn’t affected at all but after my surgery, I think it was last year when I was going to [school] to sit for my exam then all of a sudden I felt dizzy for like 20 minutes yet I was just at that junction there. So on reaching the exam room my head was empty and it was like I had never attended any lecture. So that was a retake

*Interviewer: Did you do the exam?*

***Respondent:*** I did but when the results came back it was a retake. I couldn’t handle a retake so I just cancelled my studies.

*Interviewer: You did?*

***Respondent:*** Yeah, but I will resume in August. So after my surgery, you know; this replacement. Ok, I was replaced with mechanical valves but my personal doctor told me that when I am replaced with mechanical valves I was not to do this or that anymore.

Well he told me that my diet will change and I was like, okay. On reaching there the doctor told me that I had to choose either plastic or mechanical ones but he told me that according to my age, I was [age]... Well in [year] I was in [grade] but I didn’t do my second term and that’s when I went in for an open-heart surgery. On reaching there I was told to choose either plastic or mechanical but according to my age since I was [young age], I was told to go for mechanical. The bad side of it is that I will not be able to give birth, that’s the fact.

*Interviewer: Thank you. So how have your normal lives been affected or changed because of this illness?*

***Respondent:*** I am still working but my salary was cut because I always have to come to the hospital and all the time I tell them that I am going here or there, or sometimes I feel a little dizzy and ask to rest and pause the work for a moment

## Dependence on Others

*Interviewer: Now how has it impacted your daily life? How has your daily life been affected by RHD?*

***Respondent:*** Yes. So I had to look up to my parent for each and everything, which was bad. I think I should say that I was depressed

*Interviewer: Do they appreciate, the people that you work with that you have a...*

***Respondent:*** Yeah, at least my workmates do know about my problem but if you are somewhere alone and someone needs help, you may need to go and help them yet you can’t explain to them that you are feeling like this. You really have to do something.

*Interviewer: So who buys them [medications] for you?*

***Respondent:*** Well I used to buy them because I used to work. Even my boyfriend and my dad sometimes

*Interviewer: Have you had problems accessing the drugs?*

Respondent: It’s the same thing my colleague has talked about. At times you feel you must take that medicine but because of the finances, sometimes you try to say, *“Oh God, I pray that it doesn’t harm me for just one day”*

***Respondent:*** Hmmm, at least I borrow from a friend and go and buy that medicine; no option.

*Interviewer: So who is catering for your drugs?*

***Respondent***: Auntie ***Respondent:*** My dad ***Respondent***: My husband ***Respondent:*** My parents ***Respondent:*** My dad ***Respondent:*** My boyfriend

***Respondent:*** Well that’s your husband because these days there are men whom even when you are in pain and unable to reach the hospital, he merely tells you to ask your sister or anyone to escort you, and he opts to stay behind or he tells you that he is busy with work. We have given birth to children until we stop when the man has never taken you to the hospital despite being around! He could say, *“Do you want me to look for money and at the same time go for such? Instead of looking for money you want me to sit and wait at that place where you pick drugs?”*

## Stigma

*Interviewer: Now how has it impacted your daily life? How has your daily life been affected by RHD?*

***Respondent:*** In my experience, ever since I got a heart problem, it has been kind of hard for me especially during my childhood. At least now I can sing and try to comfort myself but it was really... first of all sometimes you get comments from the society you are living in. For example, I was in high school in the boarding section but I would take tablets every day and people would see me taking them. So they would say that it is HIV and that I was just hiding it from them. So there is a way you feel. Even with education, you are studying with healthy people that you see are very happy yet for you each month you have to go to [the hospital]. In that case, there is some kind of discrimination that you feel.

*Interviewer: Do you feel judged?*

***Respondent:*** The judgment is actually not here in the hospital but outside. I remember one time my sister-in-law told my husband, *“you think nurses produce! They cannot produce; they can have like one baby in their entire life.”* Well they didn’t know my problem, they didn’t know that I was actually struggling to have one, but she was there openly commenting, *“You as a nurse can dodge the pregnancy; you know very many medications that can stop the pregnancy.”* So she was openly thinking that it was my desire yet I had my reasons which were only know by my husband. So I felt like I was being judged

***Respondent:*** They always tell me that, *“you are not even supposed to get married. You are not supposed to get married because of your RHD. No man can handle you. You won’t even give birth”.* So I feel bad whenever they say that

*Interviewer: So you say that’s it is parents and you said it’s your...*

***Respondent:*** My sister-in-law, the sister to my husband.

***Respondent:*** Friends

*Interviewer: Friends? You tell us about that*

***Respondent:*** Last year, there is a friend of mine who had an auntie who was working in

X. This lady was old and she died of cancer. Now when I asked my friend about it she was like, *“you see she had cancer and also had problems”* and then she asked me, *“Can you really give birth?”* you know those things. Now even sometimes students start looking at chronic diseases and start comparing; they can look at maybe HIV and heart disease and start saying, *“at least I would rather have...”*In fact I remember one time I came to Sister X for counseling and these people were in a room, you know like when we are out for lunch in the hall and then they bring up arguments and they are like, *“when you have a heart problem you cannot give birth; I had my friend who was inserted in a plastic valve...”*those stories. So in most cases to protect yourself you move away because you don’t want to hear what they say, but if you are caught up having your lunch with them, you are attracted to listening. So they look at us as people who are very weak in that you can be pushing the baby, that you may be pushing the baby and then your valve opens

*Interviewer: Any other group of people that judges you?*

***Respondent:*** At times when I am with my sister, you see we are only three and I am the first born, but at times when I am with her she would say, *“You are here but you are not even going to [re]produce, you are even very weak.”* Then she would afterwards start asking for forgiveness, *“I am very sorry I was just joking”* but yet it has already nagged me. So my sister really annoys me a lot.

*Interviewer: Is there any way people stigmatize you?*

***Respondent:*** That has to happen because you can study with colleagues and after school some of them get jobs, others marry or get married but you are still there! Now if you later on start communicating with each other on phone, they would say, *“What are you up to now? You are not married yet? What happened to you?”* and things like that. So you would feel... even if you try explaining to her/him. So you would still feel hurt and yet you too didn’t want it.

***Respondent:*** It has affected me a lot because from where I was staying in the beginning at my sister’s place, I fell so sick and my belly swelled up but by the time I completely healed I was slim. But in the village where I live people think I have AIDS; that has made me feel so bad. Even while working, since I work in the evenings, they are some few (who think that she has AIDS) and yet I have to swallow [take] drugs all the time and also saying that you need money for drugs all the time. So that is it; stress. So it is that issue of AIDS which has affected me most since I am [HIV] negative.

***Respondent:*** I go through the same; they detest buying me drugs and my family deserted me.

***Respondent:*** Yes, my people. Well at first, they cared for me but maybe they realized that I wasn’t healing. Now in [year] I was worse off for the whole year that I couldn’t even walk. People in the village too say that I have AIDS but I don’t care about that.

***Respondent:*** They stigmatized me since I would fall sick all the time and would always be here at [the hospital]; I think I would be admitted twice every year. Now the last time I came here and they told me that my heart needs to be operated according to its condition then, I didn’t have any money, but the people with whom I pray asked me, *“So what are you going to do now?”* I told them, *“I don’t know and I don’t even want to think about it because if I think about it I don’t know where I will get that money.”* So I then shared it with my family but since my husband had already died, they said, *“that one is sick and now she is lying to us that she has a heart problem.”* In fact when one of my aunties returned from abroad I showed her some of my medical papers and she told me, *“But now you are denying the illness (AIDS)!”* I then told her [calmly], *“If you have any hospital that you can take me to, this is what I know that I am suffering from and it is what I have brought you.”* I was however lucky that the same people with whom I pray collected money for me without even telling me about it; all they did was to give me a phone call and also got me a doctor in [country outside Uganda]where I went for my operation. Now those that used to say that I am sick were surprised to see me back in shape again and said, *“Eh! How come you are alive?”* Otherwise they say, *“Her heart is metallic”* but I don’t care about that because I am alive.

*Interviewer: Are there people that say things that are unpleasant to you?*

***Respondents:*** They do

***Respondent:*** They call us AIDS patients

***Respondent:*** They said that I turned into the ‘*Ever-sick’*

***Respondent:*** Well we talked about that and indeed it happens; they have to stigmatize us because you would have grown, not in school anymore but on medication. All the others would have given birth and their children are grown but you are still there. So they have to say, *“I wonder what happened to her? She is a heart patient. She is always on medication”* That is not a good thing; it feels so bad.

***Respondent:*** Even when we have a ceremony at home they say, *“You don’t have to invite her since she is sickly. She might even disturb us here.”*

***Respondent:*** They told me, *“ho! Your illness is even worse than for an HIV patient. An HIV patient is hopeful that they will heal but you don’t have any hope of healing.”* How would you even say such a thing to someone? So you live in such a condition of knowing that there is no healing.

***Respondent:*** Many people say, *“That one! You won’t manage her; she has (tiny) metals in her heart.”*

*Interviewer: Is that what men say?*

***Respondent:*** And also other people like the people at home and my siblings. *“That one has (tiny) metals in her heart and they are always beating; they sound like a clock. You cannot handle her. I pity her because she is going to live like that,”* they say.

***Respondent:*** The other thing they say is, “*Will you handle that one? She is so costly and if you don’t have money you won’t handle her. It’s because she is costly; her delivery will require money and everything about her requires money.”*

*Interviewer: Now as we conclude, what is it that you would like the doctors to know?*

***Respondent:*** Personally I would like to give birth and I have a boyfriend but the problem I have is my parents, especially my father. Whenever he hears that I want to give birth, he says, *“Now what man will that one give birth for? Will he live with her or it is still me who will be with that responsibility?”* He doesn’t believe and he has never at any time believed that I can give birth. You see I was here some time and Dr. X told me that I can give birth but when I went back home and shared it with my mother since I was afraid of my father. She then told him but he instead said, *“Why would she even give birth?”* That implied that he meant, *“What man can stay with her?”* So that’s my problem; I feel that I am capable with the help of God and the doctors available, but it’s my father.

***Respondent:*** I want to say something more; when we are in our communities our fellow people treat us badly because every time one says, *“that one is sick. Don’t touch that one because she is sick.”* I really feel so bothered with that thing. In fact I ask most of them, *“Did I fall sick not to heal?”*

*Interviewer: How do the family members treat you?*

***Respondent:*** Some of them counsel me while others come and support me when I am weak because sometimes I get up feeling weak and other times I am normal.

*Interviewer: What about the others; how do people treat you?*

***Respondent:*** They are supportive to me

***Respondent:*** Ever since I fell severely sick and went back, they help me because they know that I am a heart patient. I get support from them

***Respondent:*** In fact there is a family member who told me, *“that one is merely living”* but I wondered what she meant by that. But whenever people ask my husband he tells them, *“she healed”* He has never told them that I come to [the hospital] and that I pick drugs monthly. We kept that a secret to ourselves.

***Respondent:*** It is maybe my boss who used to say that he hears that I am sick but I would tell him that I am not. *“That one was about to die,”* they say, but I would tell them, *“I wasn’t dying. I am healthy.”*

***Respondent:*** What I was saying was that they stigmatize us

*Interviewer: What do they say?*

***Respondent:*** They say that a heart patient will always put you on tension; *“That one is going anytime. That one is going anytime”*

***Respondent:*** They say, *“If you are operated then you are dead. Your life will start declining slowly by slowly.”*

***Respondent:*** They judge you. In fact even if your partner was planning to marry you, they say, *“I think that man just has a lot of money; how could he marry that heart patient? Won’t the music scare her to death?”* In fact there are even times when you are somewhere and although you feel normal and comfortable, out of nowhere someone comes and tells you, *“Why don’t you take a seat! Don’t you see that you have been standing for so long?”* So one feels pity for you yet you feel okay and have no problem with your position.

*Interviewer: So they make you uncomfortable. What were you saying Respondent?*

***Respondent:*** That’s why I don’t tell anyone

***Respondent:*** Well that’s your husband because these days there are men whom even when you are in pain and unable to reach the hospital, he merely tells you to ask your sister or anyone to escort you, and he opts to stay behind or he tells you that he is busy with work. We have given birth to children until we stop when the man has never taken you to the hospital despite being around! He could say, *“Do you want me to look for money and at the same time go for such? Instead of looking for money you want me to sit and wait at that place where you pick drugs?”* Well this one (Respondent X) is lucky that her husband is able to come but mine is one of those men who can never come unless you are in a very bad condition.

*Interviewer: Are you trying to say that these men have not stood by your sides in this issue?*

***Respondent:*** At home he cares and he understands that I am sick but about going to the hospital, he never accepts.

***Respondent:*** This is what I have to say; can’t there be any other medicine they can get you for that time? For instance when one gets HIV/AIDS, they get you something meanwhile such that you are able to give birth to a healthy baby.

## Abandonment / Fear of Abandonment by Partner/Family

***Respondent:*** When I heard Respondent X talking, it reminded me of when I was newly diagnosed when my baby was four days old. When we reached the hospital, well we went through [the hospital] up to (Ward) X. So this doctor asks my husband, I think he was an intern doctor, *“is this your wife?* “and he said yes. *“How many children do you have?”* and then my husband told him one. *“How old?”* and my husband said he’s four days. Then he (doctor) was like *[in suspense]*, *“Oh my God!”* That even scared my husband. *“You should not have any other baby”* he added. I was like, *“what! We just have a baby of four days and you are now just bombing such news to my husband!”* So I wondered, *“Will this man stay with me? Is he going to leave me?* “So I was living in that fear and I was also like, *“now even the baby is four days, what if he dies? I am not breastfeeding since I have just been admitted in the hospital yet the baby is at home!”* So I lived in that fear for some time. Now this other doctor came and I think he was holding the x-ray picture and I think they didn’t know that I was in the medical field. She said *[in suspense] "what a big heart!”* So I wondered what kind of doctor this is. You see even if something is scary you should not just say it; you should say it in a calmer way. So this doctor really pissed me off. So I was later discharged and we went back home, life started normalizing and I continued with my medication but now every time my husband was telling me, *“we have to stop on one child.”* So I wondered, *“What is this man planning? Does he want to marry another wife and produce more children? Will he really be with one child?”* Well I couldn’t read someone’s mind. So I had to be on family planning for three years and I was not sure about what was going to happen but like nine months later, my husband wedded me and then I knew that he was committed irrespective of what was happening. So when he proposed a church wedding I thought that maybe now he is serious and is not going to leave me because of this. So after the wedding I proposed to him, *“can’t we have another child?”* but he completely refused and said, *“You cannot have another child*” Then I asked him, *“but how are we going to stay?” “With your condition, you can’t have another child,”* he responded. So I told him, *“I will talk to my doctors; they will assess me and see if I can have another child.”* So when I came for review I told the doctor that I wanted to have another child and she was like, *“yes you can but make sure you come for monitoring.”* Then I stopped the family planning and I delivered another child. The process of family planning was also not easy.

***Respondent:*** The problem I am facing on that is that I am not told the right time that I can get pregnant. Whenever I ask my doctor about the right time I will get pregnant he just tells me, *“you hold on I will tell you when the right time comes.”* Now of course every man wants a child; so I had a man who wanted to be introduced (to my parents) but

wanted me to get pregnant, so he left. Now I have another one but I don’t know how the case will be.

*Interviewer: How does that affect your desire to have children?*

***Respondent:*** Before I got an operation, they told me that I cannot support a baby in my womb. So they told me, *“Do not get pregnant because your condition is not good.”* By that time I had a boyfriend but I had to separate with him.

***Respondent:*** They told it to me and by that time I had a husband and my two sons. I also had my mother-in-law and they all loved me so much, but the moment they told me that they all hated me and now I stay alone.

*Interviewer: Your husband too?*

***Respondent:*** My husband said that he cannot stay with a woman who cannot give birth.

***Respondent:*** After having my first child I had an in-law who was a doctor in X Hospital. You see I had been told that my heart had a problem but in that time the echo[cardiogram] had not yet been introduced in the country. However by the time I delivered, echo had been introduced in X Hospital. I delivered very well but after giving birth I fell sick and couldn’t breathe well. In fact the person who helped me was my mother-in-law and yet I had started noticing that we were likely not to work well with the father of my child. So I requested him, *“please put me somewhere such that I give birth to my child. I have no problem with you getting someone you like.”* So we had already agreed on that and it is not that he left because of the heart disease; he had already started treating me badly, but my mother-in-law was there and took good care of me.

Now after giving birth she realized that I wasn’t breathing well so she got scared and said, *“Eh!”* but I told her that thing usually happens to me but it stops later on. She however said, *“No, not to a postpartum mother. A postpartum mother doesn’t do such things.”* She then said, *“Go to the hospital immediately.”* Then I went to X and I don’t know what they did and since I wasn’t breathing well, I don’t know what happened next when I reached the sick bed. The next time I regained my consciousness, I had already been admitted and that was after they had performed the echo on me and the results given to them. I don’t know what exactly they were told since they didn’t tell me. Maybe that too was a supplement for him to leave but at least I had already decided not to stay with him. Now when they told me that I was not to give birth again, I accepted it because I realized that I had a bad experience after giving birth and my heart wasn’t beating so fast. So I said to myself, *“I will live like that since I have this one child who will continue consoling me.”*

***Respondent:*** I go through the same; they detest buying me drugs and my family deserted me.

*Interviewer: What family deserted you; your family?*

***Respondent:*** Yes, my people. Well at first, they cared for me but maybe they realized that I wasn’t healing. Now in [year] I was worse off for the whole year that I couldn’t even walk. People in the village too say that I have AIDS but I don’t care about that.

*Interviewer: Is there any way the people at your home talk about you?*

***Respondent:*** Of course there is.

*Interviewer: What do they say?*

***Respondent:*** In fact there was a time I had an introduction ceremony but my man refused to appear because his mother had told him, *“You cannot marry that woman who cannot give birth.”*

*Interviewer: Was that on the day of the ceremony?*

***Respondent:*** Yeah, we were waiting for them but they didn’t appear. *Interviewer: Was it because you had told him that you are a heart patient?* ***Respondent:*** Yeah.

***Respondent:*** When I knew about it I just gave up and even separated with my husband. So I stayed single and lost it all.

## Financial Impacts of RHD

*Interviewer: So what did they tell you are the benefits of taking warfarin?*

***Respondent:*** The bad thing about it is that it is expensive; you have to always be with money and you don’t have to miss. Personally, take like three tabs a day and the least price most pharmacies sell it is 500 USh but others sell it 1000 USh depending on where you are.

***Respondent:*** Per tablet, but that depends on where you are. So you find it expensive and sometimes you miss when you didn’t want to.

***Respondent:*** Me I am worried about the procedures that they told me I have to go through when I get pregnant. They told me that before, I have to change from warfarin, like; they put me off warfarin to certain injections which I know are painful because I have ever gotten them. They are so painful. You can get one which is painful today and even the next day. So they told me that I have to get them for three months and I just imagine that pain! It is the same thing even in the last month when I am about to give birth; so I am just wondering. First of all, they are expensive, they are at 30,000 USh and above and every day you get one, plus the pain. So they affect me; you think a lot.

***Respondent:*** So I went to theater and delivered but after pregnancy, I get this pulmonary edema and I was coughing throughout. I was just watching my baby there helpless, he couldn’t even breastfeed. I had to start my baby on formula milk as earlier as two days. Then after that I couldn’t breastfeed all through since I didn’t have enough breast milk because Lasix was limiting my lactation. I had to put my baby on formula milk and that was an average target of 250, 000Uhs a month since my baby was taking a lot. That is the budget we have been spending. So the whole process was not easy but I went through it.

***Respondent:*** They stigmatized me since I would fall sick all the time and would always be here at Mulago; I think I would be admitted twice every year. Now the last time I came here and they told me that my heart needs to be operated according to its condition then, I didn’t have any money, but the people with whom I pray asked me, *“So what are you going to do now?”* I told them, *“I don’t know and I don’t even want to think about it because if I think about it I don’t know where I will get that money.”*

***Respondent:*** The other thing they say is, “*Will you handle that one? She is so costly and if you don’t have money you won’t handle her. It’s because she is costly; her delivery will require money and everything about her requires money.”*

*Interviewer: How much do you buy warfarin? Is it cheap?*

***Respondent:*** We buy each tablet at 500 USh

***Respondent:*** Each tablet costs 500 USh

*Interviewer: Is that affordable?*

***Respondent:*** It’s not.

***Respondent:*** Well sometimes we come here and still get stressed when they tell us about being operated. There was a time they told me that I had to be operated and that it would cost 30 million [Ugandan Shillings] and I wondered, *“Where can I even get all that money?”* When they asked me, *“can’t you think of ways of getting it?”* I told them that I don’t. Now jokingly the doctor asked me, *“don’t you have any plot of land you can sell?”* I told him, *“I would rather leave it with the children other than selling it. I don’t have 30 million [USh] and have never even seen it”* Then you would go back home and stress over that for a long time. Indeed it could take you about two months or a full month stressing over it.

***Respondent:*** [complimenting] that is very true, because I was also told that my system had to be put back to normal. If one tells you that you are to be operated for 45 million [USh] and yet you don’t even keep a million in your house and remember you have children to take care of, like I have told you that I have some children that are at the university, I am now concentrating on my children’s’ studies. So if one tells me that I am going to be operated, I would let them continue with their studies.

***Respondent:*** Personally when they told me that amount I lost my mind for a second because they told me that while I was at X Hospital. The reason why I always went to X is because my husband’s job gave us insurance but when his job was no more, Dr. X asked me to start coming here otherwise... Dr. X told me that it could cost 45 million [USh] to clean that valve and she told me a lot of other things; I was so confused. That time she called me with my husband and said, *“X’s condition is too delicate and all that.”* and when I looked at it, and besides I didn’t even have that amount. In fact you instead start just get stressed until God helped me out of that stress. I thought that if God still needs me alive, I will be. Besides, if I have ever been on oxygen support twice and even when my goiter was operated I was put in the ICU for two days and still God helped me out! So even now I am strong because if I was able to get out of the ICU, I knew that God still wants me alive. I think I won’t even be operated and besides I don’t even have the support.

***Respondent:*** I am not worried about anything ever since I found out that it was my heart which is sick. My own worry was when I didn’t know what was hurting, and maybe also having to get medicine. Since I didn’t have money I would say, *“Where am I going to get the money now?”* but I would then borrow and buy the drugs.

***Respondent:*** Drugs are so expensive, especially the initial drugs. You see there are times you come here when the pockets are not so happy and yet you are out of drugs.

*Interviewer: Have you ever skipped swallowing [taking medications[?*

***Respondent:*** You skip sometimes if you fail to get money to buy it

# Absolute Recommendations from Healthcare Providers

***Respondent:*** The bad side of it is that I will not be able to give birth, that’s the fact.

*Interviewer: That’s what they told you?*

***Respondent:*** When I heard Respondent X talking, it reminded me of when I was newly diagnosed when my baby was four days old. When we reached the hospital, well we went through [the hospital] up to (Ward) X. So this doctor asks my husband, I think he was an intern doctor, *“is this your wife?”* and he said yes. *“How many children do you have?”* and then my husband told him one. *“How old?”* and my husband said he’s four days. Then he (doctor) was like *[in suspense]*, *“oh my God!”* That even scared my husband. *“You should not have any other baby”* he added. I was like, *“what! We just have a baby of four days and you are now just bombing such news to my husband!”* So I wondered, *“Will this man stay with me? Is he going to leave me?* “So I was living in that fear and I was also like, *“now even the baby is four days, what if he dies? I am not breastfeeding since I have just been admitted in the hospital yet the baby is at home!”*

So I lived in that fear for some time. Now this other doctor came and I think he was holding the x-ray picture and I think they didn’t know that I was in the medical field. She said *[in suspense] "what a big heart!”* So I wondered what kind of doctor this is. You see even if something is scary you should not just say it; you should say it in a calmer way. So this doctor really pissed me off. So I was later discharged and we went back home, life started normalizing and I continued with my medication but now every time my husband was telling me, *“we have to stop on one child.”* So I wondered, *“What is this man planning? Does he want to marry another wife and produce more children? Will he really be with one child?”* Well I couldn’t read someone’s mind. So I had to be on family planning for three years and I was not sure about what was going to happen but like nine months later, my husband wedded me and then I knew that he was committed irrespective of what was happening. So when he proposed a church wedding I thought that maybe now he is serious and is not going to leave me because of this. So after the wedding I proposed to him, *“can’t we have another child?”* but he completely refused and said, *“You cannot have another child*” Then I asked him, *“but how are we going to stay?” “With your condition, you can’t have another child,”* he responded. So I told him, *“I will talk to my doctors; they will assess me and see if I can have another child.”* So when I came for review I told the doctor that I wanted to have another child and she was like, *“yes you can but make sure you come for monitoring.”* Then I stopped the family planning and I delivered another child. The process of family planning was also not easy

*Interviewer: What about the rest; what have you been told?*

***Respondent:*** That’s what they told me; they told me that things went wrong

***Respondent:*** My doctor told me that you can give birth but at least when you are planning to get pregnant you are supposed to come and inform them and immediately start monitoring you. She told me that if you don’t inform them and you continue with the medication you may give birth to a deformed baby. So they told me that you have to come back earlier and share with them what you are about to do and then they advise you and also take off some tablets and then introduce others [tablets].

*Interviewer: How does that affect your desire to have children?*

***Respondent:*** Before I got an operation, they told me that I cannot support a baby in my womb. So they told me, *“Do not get pregnant because your condition is not good.”* By that time I had a boyfriend but I had to separate with him.

*Interviewer: So how did that impact on your desire to have children?*

***Respondent:*** I was scared of course; I feel like I don’t want because when I hear of the impact of getting pregnant I feel like I like my life so much. I need a baby but no. Even me I can see that I am 23 and I can see that I am young but I ask myself... in fact even other people that have a similar problem like mine tell us that their doctors tell them that there is a certain age, that when you get to 30 years of age you would then be unable to

produce. So I start getting worried and just say me I have a have a brick eye and don’t look upon marriage.

***Respondent:*** Another thing affecting me when my doctor told me about the pregnancy stuff, is that is there a limit of the number of children I am supposed to give birth to? I want to know that but I don’t know.

*Interviewer: Have you ever raised that question to him?*

***Respondent:*** I did but he hasn’t yet convinced me; he says, *“you wait.”*

*Interviewer: You want a [contraceptive] method but you don’t know the method!*

***Respondent:*** Yeah

*Interviewer: Have you ever shared that with your doctor?*

***Respondent:*** Yeah, he told me to go (for one) but the method he told me, they refused to do it for me in [Hospital].

Interviewer: Why did they refuse?

***Respondent:*** I don’t know. They feared my condition. *Interviewer: What method had the doctor recommended?* ***Respondent:*** It was operating

*Interviewer: TL? [Tubal Ligation]*

***Respondent:*** Yeah

***Respondent:*** My doctor asked me, *“do you do sex with anyone?”* I told him that I do. Then he was like, *“you have to use family planning, you are not supposed to get pregnant.”* Then I was like I fear those but I am using condoms but I...

*Interviewer: Well we partly covered this but the next question was like: has the doctor ever told you the side effects of taking blood thinning medication like warfarin? I heard people mentioning some; maybe you would like to add on those. What effects have they told you about?*

***Respondent:*** They told me that if you are taking warfarin you are not supposed to be pregnant and that if you are taking warfarin you avoid accidents

*Interviewer: Now has a doctor ever told you that your heart isn’t strong enough to support a pregnancy?*

***Respondent:*** I was not told that it is weak but all they told me was, *“never get pregnant.”* Well that’s what they told me but from where I had my operation they told me

that I can get pregnant. The doctor who did the operation on me said that I can get pregnant and still live and that the hospital has a way it can maintain me. However from here, I was told never to get pregnant.

***Respondent:*** Before giving birth and you are in that age where everyone expects you to have a child, it feels bad whenever you see people with children. It feels bad that whenever you ask the doctor, *“can I try?”* and he says, *“no, your heart is still weak.”* That is really discouraging and it makes you feel stressed although you just have to live with it.

***Respondent:*** I think my heart started falling sick from the time I gave birth to my second born. That child was sickly and would get convulsions all the time and whenever she had them she would fall down and then I would start shaking and feel like my heart beating so fast. Now when the pregnancy was ready, I went for delivery but the doctor told me, *“Never get another pregnancy because your heart is sick and you might not be able to push another child. If God blesses you and you deliver properly this time, please don’t get another pregnancy.”* Well I didn’t take it so serious after giving birth but it was after about 12 years that I started feeling rapid heartbeats all the time. Whenever I went to the hospital they would tell me that I am hypertensive. It was until the doctor whom I was seeing regularly said, *“It cannot be hypertension all the time”* because the rapid heartbeats would occur every time and then I would be admitted for about three days and leave. Then within one week, I would be admitted again until he suggested, “I think you should go to Mulago Hospital such that your heart is checked.” Then I came here and confirmed that I am a heart patient.

*Interviewer: So what did the doctor tell you about having this heart disease and getting pregnant?*

***Respondent:*** He told me that if I got pregnant yet I have a heart disease, either myself or the baby, one has to die whether during delivery. So I chose to stop such that I raise these children that I have.

***Respondent:*** They told me that my heart is too weak to supply blood from me to the baby, but I didn’t listen. Like I said, I didn’t take it serious and that’s when I got pregnant with my last born. I felt so bad that now I know that I cannot get any other pregnancy

***Respondent:*** They had already told me that even before I found out that I had this illness. He told me never to get pregnant because the heart couldn’t support a pregnancy.

# Reproduction as a Balanced Risk

***Respondent:*** With pregnancy, they told us that it is a risk that you have to take. Actually, in my case, things were okay the other time; my husband was okay before I

got pregnant but when I delivered the second baby, he said, *“now you will deliver even the third one.” [Chanting from others]* So I think he thinks they were just lies in the beginning now that I had delivered the second baby. So now he is like, *“now you will have the third one and we end at that”* which is not easy since it is a risk you have to take. Recently one of my friends was telling me, *“There is a girl who died here with a six month-old baby in the womb! So, don’t deliver another baby again.”* When I told that to my husband he said, *“Let’s have the third one and we stop there.”* So it is a risk that you take.

*Interviewer: Did you know the risks it [warfarin] had to the baby?*

**Respondent:** Yes, I knew

*Interviewer: Thank you so much. Would you be willing to use birth control methods when you are taking warfarin?*

***Respondent:*** If they can prevent me from getting pregnant, because I know that I am not supposed to get pregnant when I am on warfarin because it is dangerous.

***Respondent:*** I am willing if I am given the best option. I hear of IUDs, well I don’t know about them but when they give me the best option, I can.

***Respondent:*** I would be willing to take a family planning method if I am on warfarin but now that I am not on, that’s why I am saying that I would rather deliver and finish and then do a BTL (bilateral tubal ligation) because even condoms are not reliable especially in a family; some men really don’t like them and it is inconveniencing so sometimes you may do it without it [condom]. You may not even be able to keep it in the house there and then. So I think if I am on warfarin, the best family planning method I can go for is a permanent one such that I know that I am not having any other child.

*Interviewer: What about the others; how does knowing that the heart is weak influenced whether your interest in family planning? Respondent are you on any method?*

### Respondent: No.

*Interviewer: Would you have wanted one?* ***Respondent:*** At the moment no *Interviewer: Why?*

***Respondent:*** Because I am not dating anyone to make me feel like am at risk

*Interviewer: How has knowing the side effects of warfarin affected your view and decision to get pregnant or not and to use family planning?*

***Respondent:*** You decide to be on family planning because your life is priority but it’s not that you don’t want to get pregnant mainly because of warfarin. Of course warfarin is

an issue but I cannot say that it is the main reason that can stop me from using family planning. The heart; because they told me that my heart is weak, it is the main reason why I can decide to use family planning. Of course the issue of warfarin is there but I cannot say that bleeding is the reason I should go in for family planning. If that’s the case then it would appear like I have foregone the main reason and precede the issue of the medicine.

***Respondent:*** I was told that if I don’t swallow [take] warfarin I would get a stroke and the blood and goes to the brain

*Interviewer: So what did the doctor tell you about having this heart disease and getting pregnant?*

***Respondent:*** He told me that if I got pregnant yet I have a heart disease, either myself or the baby, one has to die whether during delivery. So I chose to stop such that I raise these children that I have.

***Respondent:*** [complimenting] that is very true, because I was also told that my system had to be put back to normal. If one tells you that you are to be operated for 45 million [USh] and yet you don’t even keep a million in your house and remember you have children to take care of, like I have told you that I have some children that are at the university, I am now concentrating on my children’s studies. So if one tells me that I am going to be operated, I would let them continue with their studies.

*Interviewer: And then leave the money for the children!*

***Respondent:*** Yeah, that is a lot of money.

***Respondent:*** Yeah, I have gambled like that up to now. Now this one is about to make six years old and I am still using it [safe-days method]. Now I don’t know if... I don’t know. You see when I came here I had been told that I would be stopped completely from conceiving. While I was still at X I was to be stopped completely (using tubal ligation) from conceiving but when I came here, since there are many health workers and each of them saying something different and also since I was in a critical condition, maybe they forgot about it. Also I feared getting back to the theater for a second time. That is my condition now.

**Patient Desire for Fertility**

***Respondent:*** When I heard Respondent X talking, it reminded me of when I was newly diagnosed when my baby was four days old. When we reached the hospital, well we went through [the hospital] up to (Ward) X. So this doctor asks my husband, I think he was an intern doctor, *“is this your wife?* “and he said yes. *“How many children do you*

*have?”* and then my husband told him one. *“How old?”* and my husband said he’s four days. Then he (doctor) was like *[in suspense]*, *“Oh my God!”* That even scared my husband. *“You should not have any other baby”* he added. I was like, *“what! We just have a baby of four days and you are now just bombing such news to my husband!”* So I wondered, *“Will this man stay with me? Is he going to leave me?* “So I was living in that fear and I was also like, *“now even the baby is four days, what if he dies? I am not breastfeeding since I have just been admitted in the hospital yet the baby is at home!”* So I lived in that fear for some time. Now this other doctor came and I think he was holding the x-ray picture and I think they didn’t know that I was in the medical field. She said *[in suspense] "what a big heart!”* So I wondered what kind of doctor this is. You see even if something is scary you should not just say it; you should say it in a calmer way. So this doctor really pissed me off. So I was later discharged and we went back home, life started normalizing and I continued with my medication but now every time my husband was telling me, *“we have to stop on one child.”* So I wondered, *“What is this man planning? Does he want to marry another wife and produce more children? Will he really be with one child?”* Well I couldn’t read someone’s mind. So I had to be on family planning for three years and I was not sure about what was going to happen but like nine months later, my husband wedded me and then I knew that he was committed irrespective of what was happening. So when he proposed a church wedding I thought that maybe now he is serious and is not going to leave me because of this. So after the wedding I proposed to him, *“can’t we have another child?”* but he completely refused and said, *“You cannot have another child*” Then I asked him, *“but how are we going to stay?” “With your condition, you can’t have another child,”* he responded. So I told him, *“I will talk to my doctors; they will assess me and see if I can have another child.”* So when I came for review I told the doctor that I wanted to have another child and she was like, *“yes you can but make sure you come for monitoring.”* Then I stopped the family planning and I delivered another child. The process of family planning was also not easy.

***Respondent:*** Yes, he knows because I have to tell him. Yes, he told me that he will patiently wait but it is also killing me because I want a baby.

*Interviewer: How has the information the doctor gave you impacted on your desire to have children?*

***Respondent****:* It gave me some hope because initially... You see every woman, now I am grown and I am in a serious relationship although (staying) alone here. So you get hope. I have a friend called X who was operated and she lives in X. She gave birth after one year of her operation and then they separated with... well because she was young and still in Ordinary level and the boy who was dating her was a student. Now X usually tells me, *“however much I separated with this guy, my kid gives me hope. Sometimes I can be on bed feeling pain and then my kid comes and asks me [in a kid’s voice]– mummy, are you sick-? So I would feel like the person is caring.”* So when the doctor gave me that information, because at that time when I had come for my injection my heart was beating so fast and we didn’t know the cause but they were like, because I

am growing my valve bends and the pressure increases since they are narrowed somehow because of much blood, the pressure is high. So when the doctor told me that it is possible (to have a baby) I got some hope. So in whatever I do I do it with hope; I don’t care but if I have at least one kid, it would be enough.

*Interviewer: So after having gotten that information, how has that information impacted on your desire to have children? How has that information you were given about side effects impacted your desire to have children?*

***Respondent:*** To me it has not because I decided. Well when I was told that I won’t have any child, I was kind of low but I never bothered because I was still young then. But now I decided that when I get to maximally 30 years of age, I will get pregnant and I will endure each and everything; whatever comes up. I don’t know, but I am ready to take the risk

***Respondent:*** They scare me but I realized that if you go through the right procedures, and also I have seen people with whom I share similar problem, they went through the right procedure. I feel I can take a risk for one [child].

***Respondent:*** I would wish us not to be judged in case they see any of us here pregnant, like *[in a tougher tone]*, *“why did you get pregnant?”* They should accept that we are also human beings and have decisions. I saying that someone told me *[in an accusing manner]*, *“that woman died, why did she get pregnant? Now she died with a baby in the womb!”* So we should not be judged because we are also human beings. Much as we have this in our body, we have desires, we want children and we want families. So when it happens we should be accepted and be managed and given the utmost support that we need instead of being questioned why we got pregnant because I cannot remove the pregnancy at that time and besides, if I am pregnant I really wanted it! I took it and I knew it was a risk but when it happens I shouldn’t be pinned and judged as to why it happened because any of us here can come at one time while pregnant but when it happens they should say *[in a calmer tone] “you are pregnant? Then let’s try our best and see how you go through it.” [supportively]*

*Interviewer: So how have they impacted your decision to get pregnant?*

***Respondent:*** I am still insisting; I want a child. We shall see from there. [Others laugh] ***Respondent:*** It never changed when they told me. Still I will try with God as my savior. I will try giving birth even if it is one child.

*Interviewer: How has knowing the side effects of warfarin affected your view and decision to get pregnant or not and to use family planning?*

***Respondent:*** Well if I have a husband and have been told about warfarin and family planning, I can bear with family planning; *injecta-plan* and then continue taking that

medicine because it is what controls the flow of your blood. Besides, life is our first priority.

***Respondent:*** I would like to have my own child, so it hasn’t affected me

*Interviewer: What about your desire to get children; has it been affected now that you know that your heart cannot support a pregnancy?*

***Respondent:*** If I am not to get one, then it will be hard for me.

***Respondent:*** Of course one would feel bad. Everyone has to give birth at least once

***Respondent:*** Not having a child is so hurting!

**Patient Beliefs about Limitations of Contraceptives/Misunderstandings about Contraceptives**

*Interviewer: What did you use?*

***Respondent:*** I was using IUD but it was like I was risking because everything I read about it, it is category four and I have to weigh the risk, because it causes a risk of endocarditis. I couldn’t go for hormonal because of the risk of coagulation. Practically I didn’t have an option apart from barrier methods which are also not convenient at all. So I had to go for an IUD for all those years.

*Interviewer: What were you fearing?*

***Respondent:*** Family planning methods. I feared that they would bring me a lot of blood. So I feared and that’s why I conceived

*Interviewer: So have you ever been offered a birth control method? Have they given you contraceptives?*

***Respondent:*** Me I used an IUD twice but still I know that I am risking because when I read about it, they were saying that there is a risk of infective endocarditis, so I make sure I don’t miss my Benzathine monthly and I let my husband know about the risk and how it can come about.

***Respondent:*** Personally, the knowledge I got is that in most cases our nurses do a lot of things because they are freer than the doctors. Now I got information from one of our nurses and she told us that with family planning, the things that they put in your body are bad and that they can cause clotting of the blood.

***Respondent:*** What they told me while I was in [country outside of Uganda], they told us that family planning is not good if you are on medication or if you are suffering from heart disease; it is very dangerous since it has many side effects unless you use condoms or you abstain

*Interviewer: Do you mean that family planning is not there?*

***Respondent:*** Personally I was refused to use it. Since my blood pressure had risen, they told me that if I added contraceptives it would further increase. My doctor told me that if I am to use contraceptives it would increase my blood pressure and since my heart is sick, it could affect my heart. He told me that it is better if I used safe days.

Even with the coil, I don’t wear watches. The coil has a metal which might make me react. So he told me about the safe days. Actually I was using that method when I got that baby. I got that baby when the other kid was six years old. I spent those six years using the safe-days method. He told me that if I used contraceptives there would be higher risks of increased blood pressure and a faster heartbeat and all similar issues.

***Respondent:*** For me I want to be enlightened about the family planning; the tablets or pills, because I was told that when you take pills they affect the baby and that’s why these days many people are giving birth to babies with heart holes. Others come out when they have big heads. That is the effect of family planning

# Control of Reproduction by External Actors

## Control of Reproduction by Male Partners

***Respondent:*** In my case, things were okay the other time; my husband was okay before I got pregnant but when I delivered the second baby, he said, *“now you will deliver even the third one.” [Chanting from others]* So I think he thinks they were just lies in the beginning now that I had delivered the second baby. So now he is like, *“now you will have the third one and we end at that”* which is not easy since it is a risk you have to take. Recently one of my friends was telling me, *“There is a girl who died here with a six month-old baby in the womb! So, don’t deliver another baby again.”* When I told that to my husband he said, *“Let’s have the third one and we stop there.”* So it is a risk that you take.

***Respondent:*** The number of children also affects the use birth control methods. Now I have two children and I would go for a permanent method like a BTL but I can’t because then my husband would know that it is done and we couldn’t have any other child. And then, I would not say I would rely on condoms or withdrawal methods because I know that it could make me pregnant. So sometimes you take the risk and use the one which

you know that has effects. So that is affected by the number of children. If I had the number which I want, I would completely cut it off.

*Interviewer: How many children did you have in plan to have before you were told?*

***Respondent:*** Personally getting children was to depend on how my husband was to treat me. I didn’t have any specific plan on how many children I was to have but I thought that if he treated me well and also cared well for me; maybe then I would say that I would like four children. You can plan that you will have four children yet there is no money to take care of them. It was to depend on the situation.

***Respondent:*** The problem is that I am with someone who is still in school and there is no way I can commit to get pregnant yet he wouldn’t be able to take care of that pregnancy. So I have to be on family planning such that nothing separates us if we have sexual intercourse. If a time reaches and he is done with his studies, I can then give birth.

***Respondent:*** The other thing is that if you are husband and wife, the man might ask for children by all means.

## Control of Reproduction by Physicians

***Respondent:*** The problem I am facing on that is that I am not told the right time that I can get pregnant. Whenever I ask my doctor about the right time I will get pregnant he just tells me, *“you hold on I will tell you when the right time comes.”* Now of course every man wants a child; so I had a man who wanted to be introduced (to my parents) but wanted me to get pregnant, so he left. Now I have another one but I don’t know how the case will be.

## Control of Reproduction by Others

*Interviewer: Do you feel judged?*

***Respondent:*** The judgment is actually not here in the hospital but outside. I remember one time my sister-in-law told my husband, *“you think nurses produce! They cannot produce; they can have like one baby in their entire life.”* Well they didn’t know my problem, they didn’t know that I was actually struggling to have one, but she was there openly commenting, *“You as a nurse can dodge the pregnancy; you know very many medications that can stop the pregnancy.”* So she was openly thinking that it was my desire yet I had my reasons which were only know by my husband. So I felt like I was being judged.

# Areas for Improvement in the Healthcare System

***Respondent:*** A certain doctor told me that I might get fibroids in the stomach when I reach like 25 and above years before getting pregnant. So I am as well scared of that.

*Interviewer: So you want to reach 25 when you have a baby?*

***Respondent:*** No, I want to have a family but that has stopped me. I don’t know when it will happen.

*Interviewer: So you want that information to be clear!*

***Respondent:*** Yeah

***Respondent:*** Another thing affecting me when my doctor told me about the pregnancy stuff, is that is there a limit of the number of children I am supposed to give birth to? I want to know that but I don’t know.

*Interviewer: Have you ever raised that question to him?*

***Respondent:*** I did but he hasn’t yet convinced me; he says, *“you wait.”*

***Respondent:*** My doctors never told me anything concerning pregnancy but the thing is I just get scared and am like, when I get pregnant wont I get so tired while producing and then I die? I really have very many...

*Interviewer: So what are the barriers people with RHD get when they are obtaining birth control treatments or therapies? What are the hindrances that you are facing as you try to access them?*

***Respondent:*** The family planning providers out there don’t have knowledge about the best methods. So if it was not that I am a nurse and know how to read and know what is good for me, it wouldn’t be well. Even sometimes the doctors that we see here don’t have the best options; I remember one time my doctor recommended Mirena. Mirena is an IUD which is hormonal but when I went specifically to a specialist in gynecology, she is the one who told me, *“Don’t go for Mirena because it is also hormonal.”* So sometimes even the doctors who see us here don’t know what could be best for us. So the knowledge regarding the method that we need is not there.

*Interviewer: Thank you for sharing that information. So for those who got scared, were you offered birth control methods when they told you about the side effects of warfarin?*

### Respondent: No

***Respondent:*** Not yet

*Interviewer: Others, have you been given enough information? Do you feel like you have been given enough information about family planning?*

### Respondents: No

*Interviewer: So what do you really want to know about family planning? Where do you feel there are gaps?*

***Respondent:*** I want to know whether, I hear of the contraceptive pills, if I take such family planning method it won’t affect my warfarin. If it doesn’t affect it then why wouldn’t I apply it?

***Respondent:*** For me I want to be enlightened about the family planning; the tablets or pills, because I was told that when you take pills they affect the baby and that’s why these days many people are giving birth to babies with heart holes. Others come out when they have big heads. That is the effect of family planning

***Respondent:*** The message I would wish to be passed to all of us and not just me in particular is the how the different family planning methods affect other medications that we are taking not only warfarin, and also what is the best choice and what risks do we incur when we go for them. So I wish we could have that package and access it and we know which one is the best.

*Interviewer: So is there any information that you lack about RHD and being pregnant? Is there any missing bit where you want to be beefed up?*

*Interviewer: What is it that you want the doctors to pass on?*

***Respondent:*** What should I be eating? In my food nutrition, what should I be eating?

***Respondent:*** I want to know the exact right time I should get pregnant.

***Respondent:*** Now that I have gone through the pregnancy with the disease, the biggest controversy that I got when I was pregnant was the mode of delivery. I wish that one could be really clearly explained. They were very many doctors who had different ideas. Others told me to go for a forceps delivery, another one telling me vaginal delivery and another one telling me Caesarean delivery if the pregnancy is normalized well. So I wish that one would be re-planned such that you know that if you get pregnant, this will be the mode of delivery. Otherwise I ended up with the Caesarean which I knew had risks with my disease but I wish we could get some information about it such that by the time someone gets pregnant they know how they are going to deliver that pregnancy.

***Respondent:*** Let me call it a request; if possible these doctors should once in a while, remember we are in that age of reproduction. I wish they could provide room for counseling of couples. For example, we have RHD and when you come with someone who is serious or your husband or boyfriend and maybe they talk to the two of you (as a couple).

***Respondent:*** I would wish us not to be judged in case they see any of us here pregnant, like *[in a tougher tone]*, *“why did you get pregnant?”* They should accept that

we are also human beings and have decisions. I saying that someone told me *[in an accusing manner]*, *“that woman died, why did she get pregnant? Now she died with a baby in the womb!”* So we should not be judged because we are also human beings. Much as we have this in our body, we have desires, we want children and we want families. So when it happens we should be accepted and be managed and given the utmost support that we need instead of being questioned why we got pregnant because I cannot remove the pregnancy at that time and besides, if I am pregnant I really wanted it! I took it and I knew it was a risk but when it happens I shouldn’t be pinned and judged as to why it happened because any of us here can come at one time while pregnant but when it happens they should say *[in a calmer tone] “you are pregnant? Then let’s try our best and see how you go through it.” [Supportively]*

***Respondent:*** For me it’s just that, I don’t know; is it possible if we had a doctor, from our doctors here at Uganda Heart institute, who can go for further training about the process of delivery. Because I remember they were telling us that it is not good to deliver from those small clinics because many patients that have lost their lives. There is a friend who went and had an operation but because she ran away from home, the husband didn’t tell them that the girl was operated. Well I don’t know much because it was just the auntie trying to advise me but, they (doctors) tampered with the upper part which resulted into her death. Then also I have a friend who lives in X; she got the first pregnancy and she didn’t care and then the baby died in her womb at seven months but then when she came here, they told her to go to the delivery ward. When she reached there all those doctors, because they were supposed to give her an injection for the baby (fetus) to come out, they were like, *“you are so delicate, and we can’t touch you.*

*We may even kill you as well because the baby is already dead.”* So I wish we had our doctors here trained such that there is a place where RHD patients are advised to deliver from. If they can always be there because it is not regular that you are going to give birth every day; it is something that happens once. They should be there to at least show guidance in the delivery because X told me that while she was delivering, since she was still young she was put something here *[shows interviewer]* and then she didn’t push the baby herself. She would push but again with some assistance since she had created a good relationship with the sister (nurse). Therefore the sister was caring.

*Interviewer: So you need a special doctor to handle!*

***Respondent:*** To at least be there for guidance

***Respondent:*** I delivered my baby from X but at the time of delivery, even the doctors were a little bit confused. They were asking me, *“Are you going to bring your anesthesiologist from [Uganda] Heart Institute?”* It was like there was some bit of confusion but then they needed to bring out the baby so quickly because the baby was in danger. So like she has said, comprehensively this issue must be looked at and they incorporate right from before getting pregnant, pregnancy, to delivery and at least a gynecologist input should be there up to the time the baby is out.

*Interviewer: Now as we conclude, what is it that you would like the doctors to know?*

***Respondent:*** Personally I would like to give birth and I have a boyfriend but the problem I have is my parents, especially my father. Whenever he hears that I want to give birth, he says, *“Now what man will that one give birth for? Will he live with her or it is still me who will be with that responsibility?”* He doesn’t believe and he has never at any time believed that I can give birth. You see I was here some time and Dr. X told me that I can give birth but when I went back home and shared it with my mother since I was afraid of my father. She then told him but he instead said, *“Why would she even give birth?”* That implied that he meant, *“What man can stay with her?”* So that’s my problem; I feel that I am capable with the help of God and the doctors available, but it’s my father.

*Interviewer: So how would you like the doctors to help you with that?*

***Respondent:*** I don’t know if they can speak to him

*Interviewer: With your father?*

***Respondent:*** Yeah, that’s what I want.

***Respondent:*** What I would request the doctors is that if possible they should search an easier drug for us which is not like warfarin which is so hard. We need a drug where even if you get pregnant it is not affected much such that we could give birth

***Respondent:*** Now that I am not yet ready to give birth, I request to start on family planning and I request the doctors to help me with that. The other thing is, well I don’t know if this question is related to the topic; I would like them to help us with that Benzathine. I wish there were Benzathine tablets otherwise that monthly Benzathine injection! In fact we no longer have buttocks! There is no more because you are injected every month and you find the whole buttocks so swollen. They should help us get another type of treatment.

*Interviewer: Is there anything else about this topic that you would like to share with us?*

***Respondent:*** Well about that injection Respondent X has talked about, I had a bad experience with it in the beginning but ten years after my operation, I ended up returning almost every month although I had been told to return every three months. The doctor then told me, *“now we are going to put you back to our injection!”* You see I was happy because of not getting that injection for all those years but because of that problem, I accepted. Besides, after getting that injection, except on rare occasions, I can take hold until the time I am told to return and then return at that unless I return due to some other issues. Otherwise the infections reduced a bit.

***Respondent:*** The other thing is that they should get us counselors because there are times we get stressed. The hospital should get us counselors such that if one is stressed they can come and speak to someone and feel better.

***Respondent:*** What you have said is true; the health workers that give us the drugs never tell us the side effects of those drugs. I have never been told what side effects I would get in what I swallow [take] or in the injections that I get.

***Respondent:*** Sometimes they get busy and I think it is what stops them. We don’t take time to explain everything to them about we feel because there are always many patients.

***Respondent:*** S/he doesn’t have a lot of time for you. Except there was a time when one of the patients was in a very bad condition, I think it is such people that are given time. However if he checks you and you are not so badly off, you get straight to the point: he asks you how you feel and then you tell him since that would be your scheduled appointment. Then after telling him, he prescribes drugs for you. In fact after prescription, Madam X is always nearby; she would get your paper and then someone else gets in.

***Respondent:*** To add to that, if that situation arises where the man doesn’t accept it, you can bring him the health workers to counsel him. For instance I think my husband never used to find my condition so serious but when Dr. X called him, he became keener. In fact from then he started saying (to the children), *“don’t allow your mother to wash clothes.”* Personally I think that whenever a man listens to a third party, of course he might read the papers and doubt them but if someone counsels him, he can calm down and start devising other means as a person. I think whenever my husband read it in papers he couldn’t understand it very well. It was until Doctor X called him and spoke to him; she asked him, *“are you aware that your wife is sick?”* to which he replied that he was told that I am sick. Then she asked him, *“how did you understand her illness?”* and he said, *“They said that her heart is sick but I don’t know what.”* Then she told him the problem with my heart and later I noticed that he took it so seriously because he started saying, *“why do you allow your mother to wash clothes?”* So when I did something I could see that he cared much more than before. So I think that if that man is sat down and spoken to by health workers, there is a better impact than when you tell him yourself.

***Respondent:*** Well we were talking about the instance where the health workers have told you that you are unable to give birth but the man wants children. That’s when I brought in that issue; men listen more to words from the health workers than from us. He might not believe you but when the doctor explains to him he would believe him/her.

*Interviewer: So the counseling comes in at that point!*

***Respondent:*** Yeah, the counseling comes in at that point where the man is counseled about pregnancy such that he understands that that one child should be enough or if not, like she said; he can try outside (the marriage).

**‡ Notes on coded comments:** This supplementary material consists of comments made by focus group participants grouped by codes considered relevant by our research team. The data from all three focus groups has been combined. All demographic information, locations, names, and other potentially identifying information including respondent number have been removed from this document. Original focus group transcripts containing the redacted information are available upon request to the authors pending approval from appropriate institutional authorities.
